# Construction and characterization of a genomic BAC library for the *Mus m. musculus *mouse subspecies (PWD/Ph inbred strain)

**DOI:** 10.1186/1471-2164-6-161

**Published:** 2005-11-16

**Authors:** Petr Jansa, Petr Divina, Jiří Forejt

**Affiliations:** 1 Institute of Molecular Genetics, Academy of Sciences of the Czech Republic and Center for Applied Genomics, Vídeňská 1083, CZ-142 20, Prague 4, Czech Republic

## Abstract

**Background:**

The genome of classical laboratory strains of mice is an artificial mosaic of genomes originated from several mouse subspecies with predominant representation (>90%) of the *Mus m. domesticus *component. Mice of another subspecies, East European/Asian *Mus m. musculus*, can interbreed with the classical laboratory strains to generate hybrids with unprecedented phenotypic and genotypic variations. To study these variations in depth we prepared the first genomic large insert BAC library from an inbred strain derived purely from the *Mus m. musculus*-subspecies. The library will be used to seek and characterize genomic sequences controlling specific monogenic and polygenic complex traits, including modifiers of dominant and recessive mutations.

**Results:**

A representative mouse genomic BAC library was derived from a female mouse of the PWD/Ph inbred strain of *Mus m. musculus *subspecies. The library consists of 144 768 primary clones from which 97% contain an insert of 120 kb average size. The library represents an equivalent of 6.7 × mouse haploid genome, as estimated from the total number of clones carrying genomic DNA inserts and from the average insert size. The clones were arrayed in duplicates onto eight high-density membranes that were screened with seven single-copy gene probes. The individual probes identified four to eleven positive clones, corresponding to 6.9-fold coverage of the mouse genome. Eighty-seven BAC-ends of PWD/Ph clones were sequenced, edited, and aligned with mouse C57BL/6J (B6) genome. Seventy-three BAC-ends displayed unique hits on B6 genome and their alignment revealed 0.92 single nucleotide polymorphisms (SNPs) per 100 bp. Insertions and deletions represented 0.3% of the BAC end sequences.

**Conclusion:**

Analysis of the novel genomic library for the PWD/Ph inbred strain demonstrated coverage of almost seven mouse genome equivalents and a capability to recover clones for specific regions of PWD/Ph genome. The single nucleotide polymorphism between the strains PWD/Ph and C57BL/6J was 0.92/100 bp, a value significantly higher than between classical laboratory strains. The library will serve as a resource for dissecting the phenotypic and genotypic variations between mice of the *Mus m. musculus *subspecies and classical laboratory mouse strains.

## Background

PWD/Ph is a highly inbred strain currently at 81 generations of brother × sister matings. It originated from the *Mus m. musculus *mouse subspecies by systematic inbreeding of a pair of wild mice trapped in 1972 [1,2]. The mouse subspecies *M. m. musculus *and *M. m. domesticus *diverged from their common ancestor about 300 thousand years [3] to 1 million years ago [4] and at present they display signs of incomplete reproductive isolation [5-7]. As a consequence of the interrupted gene flow between both subspecies, the mice of the PWD/Ph strain exhibit a high degree of DNA polymorphisms and a broad range of phenotypic differences when compared to classical laboratory strains [2,8]. Because of this unique feature, the PWD/Ph inbred strain has been nominated among 15 mouse strains, the genomes of which are being resequenced using high-density oligonucleotide array technology by Perlegen Sciences, Inc. [9]. Moreover, PWD/Ph serves as the chromosome donor strain in construction of a set of C57BL/6-Chr^PWD ^chromosome substitution strains (Gregorova, Forejt and coworkers, in preparation).

Bacterial Artificial Chromosome (BAC) genomic libraries are source of large genomic DNA insert clones for sequencing projects, physical mapping and isolation of intact genes [10,11]. Although BAC clones may carry large inserts of genomic DNA (up to 200 kb) they display low rate of *de novo *rearrangements and are easy to handle. These features are in strong favor of the BAC libraries over the Yeast Artificial Chromosome (YAC) libraries, which can contain up to 60% of chimeric clones [12]. Transgenic mice can be generated using BAC clones to examine candidate genes in context of all regulatory DNA elements required for their function and the phenotype of a mutant mouse can be rescued by BAC transgenesis [13,14]. Moreover a targeted modification at exact positions within a genomic BAC clone can be introduced by recombineering [15,16].

Here we report construction and characterization of the PWD/Ph BAC library, the first genomic library of the *Mus m. musculus *mouse subspecies. This library together with the upcoming panel of chromosome substitution strains will serve as a tool for analysis of complex traits by taking advantage of the evolutionary divergence between the two closely related mouse subspecies.

## Results and discussion

### Construction of the PWD/Ph-BAC library

The BAC library was prepared by cloning the *Eco*RI-partially digested genomic DNA from the spleen of a PWD/Ph female mouse in the vector pBACe3.6. Female DNA was chosen to gain an unbiased representation of the X chromosome in the library. The primary clones were picked and arrayed in 377 individual 384-well plates. The library consists of two segments containing 54 144 and 90 624 clones, respectively. Together 144 768 primary clones were arrayed on eight high-density nylon membranes (18 342 clones in doublets per membrane). The high-density membranes were utilized in subsequent hybridization experiments.

### Average insert size of the library

**Figure 1:**
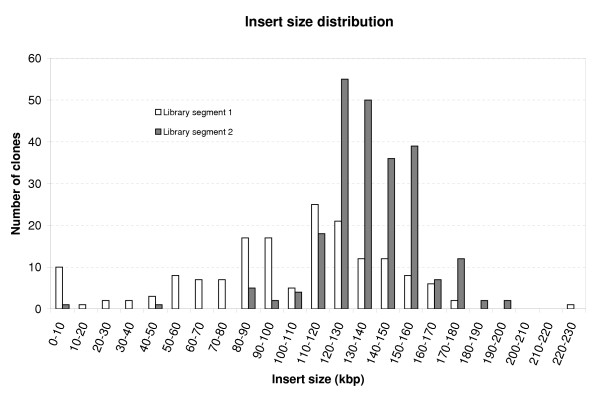
**Insert size distribution in two segments of the PWD/Ph BAC library**. The segment 1 (□) represents 37.4% of the clones and its average insert size was 101.1 kb (SD ± 21.4). The segment 2 (■) represents 62.6% of the clones and its average insert size was 129.5 kb (SD ± 14.7). The average insert size of the entire library was 120 kb.

The average insert size of the library was determined on a set of 400 randomly selected BAC clones. DNA samples were prepared from 164 and 236 BAC clones from the library segments 1 and 2, respectively, and subjected to *Not*I restriction analysis. The products of the digestion reactions were resolved by pulsed-field gel electrophoresis (PFGE) along with the high molecular weight markers. The average insert size for the first and the second library segment was 101.1 kb (SD ± 21.4) and 129.5 kb (SD ± 14.7), respectively (Figure 1). In the first and second library segments 6% and 1.2% clones were observed without insert, corresponding to 97% of insert-containing clones for the entire library. Estimation of 6.7-fold redundancy of the library was based on the average insert size (120 kb) and 2.6 × 10^9 ^bp size of the mouse genome.

### Library screening and BAC end sequencing

A probability to find any given unique sequence in the library is 99.85%, according to the published formula (P = 1 - e^N.ln(1-I/GS)^, where P is probability, N is number of clones, I is insert size, and GS is size of genome) [17]. To further assess the genome redundancy and possible cloning bias of the library experimentally we performed a screening of the library with 7 single-copy gene probes. The probes were designed to amplify PCR products on the PWD/Ph genomic DNA template (Table 1). Seven probes detected in total 48 positive clones by hybridization on 8 high-density library membranes, 4 to 11 clones for each individual probe. The average number of clones recognized by a single probe was 6.9, in good accordance with the assessment of the library redundancy based on the average insert size.

**Table 1:** Hybridization of single-copy gene probes on high-density membrane

Gene/Primer	GenBank accession/Primer sequence	Genomic position^1^	Length	Positive clones
*Mash*	NM_008554			
mMashCgi-F	ACCCGGTTCCTCGCGAGCACTTTTC	chr7:130,673,330	358 bp	5/48
mMashCgi-B	AGCGCAGCGTCTCCACCTTACTCAG	chr7:130,672,998		
*Adseverin*	NM_009132			
CpG-Ads-1F	TCTTGGAGGGTCATACTCATT	chr12:35,153,275	516 bp	7/48
CpG-Ads-1R	GCAGCTCAAAATAATTACGAC	chr12:35,152,780		
*Igf2r*	NM_010515			
Igf2r-H4F	TCAGAACACTGGTGAGCAGTGGG	chr17:12,150,732	244 bp	11/48
Igf2-H4R	GAGGGTAGGATTCCGTTGCAAGG	chr17:12,150,509		
*Tbp*	NM_013684			
	* probe derived from tbp-1942 clone	chr17:14,324,440	1085 bp	4/48
		chr17:14,334,524		
*Usp26*	NM_031388			
Usp26-A	AATGTAACGAAGGGAGAAGTG	chrX:44,101,298	206 bp	3/48
Usp26-B	AGGCTTTGCCTTCTTATCGAG	chrX:44,101,113		
*Xist*	AY618354			
mXistF	AGTGGGTGTTCAGGGCGTGG	chrX:94,885,925	293 bp	11/48
mXistR	CTATCCCCTAGTCCTCTGCGG	chrX:94,885,652		
*Tex13*	NM_031381			
Tex13 pub-1F	ACCAGAGTTGGGAACAACTAA	chrX:130,816,501	220 bp	7/48
Tex13 pds-1R	CTGTTGTAGAGGGTAGAGGTT	chrX:130,816,302		

^1 ^mm5 assembly, UCSC

To characterize the inserts of the PWD/Ph BAC library at the DNA sequence level we sequenced and manually edited 87 BAC ends from 47 BAC clones (total 38,339 nucleotides). The BAC end sequences (BESs) were masked for repeats and aligned on the C57BL/6J mouse genome. BES pairs of 29 BAC clones mapped to unique positions in the B6 genome on the opposite DNA strands within the distance up to 200 kb (Additional file 1). The mapping allowed us to estimate the average insert size of the BAC clones based on their locations on the B6 genome as 127 kb, which was slightly higher estimate than the average insert size acquired by restriction analysis (120 kb). These values corresponded well with the average insert size calculated for another set of clones recovered by the library screening described above (Table 2). A BES pair belonging to the clone 307-9O mapped to two distinct chromosomes. Whether it represents a chimeric insert or a chromosomal rearrangement in the PWD/Ph genome remains to be determined by fluorescence in situ hybridization (FISH) analysis. For each of the additional 13 BAC clones we found unambiguous positions for only one BES of a pair. Mapping of remaining 14 BESs was prevented by a high content of repetitive elements.

**Table 2:** BAC end sequences of the positive clones mapped on the C57BL/6J genome

Gene	Probe position	BAC clone/primer	BES position	Strand	Insert size (bp)	
					
					mapped	PFGE
*Mash*	chr7:130,672,998	327-5I/T7	chr7:130,587,753	+	132,317	145,000
		327-5I/SP6	chr7:130,720,069	-		
*Ads*	chr12:35,152,780	262-11G/SP6	multiple hits		n.d.	105,000
		262-11G/T7	chr12:35,192,443	-		
		266-2E/SP6	chr12:35,090,782	+	109,399	115,000
		266-2E/T7	chr12:35,200,180	-		
*Igf2r*	chr17:12,150,509	279-5I/SP6	chr17:12,057,702	+	125,000	120,000
		279-5I/T7	chr17:12,182,701	-		
		282-5D/T7	chr17:12,028,446	+	144,525	145,000
		282-5D/SP6	chr17:12,172,970	-		
		245-11P/SP6	chr17:12,000,500	+	186,822	195,000
		245-11P/T7	chr17:12,187,321	-		
*Tbp*	chr17:14,324,440	297-2I/T7	chr17:14,291,556	+	147,925	145,000
		297-2I/SP6	chr17:14,439,480	-		
*Usp26*	chrX:44,101,113	293-21P/T7	chrX:44,086,068	+	184,994	190,000
		293-21P/SP6	chrX:44,271,061	-		
*Xist*	chrX:94,885,652	269-9H/SP6	chrX:94,817,775	+	150,314	160,000
		269-9H/T7	chrX:94,968,088	-		
		255-3L/T7	chrX:94,756,622	+	134,724	145,000
		255-3L/SP6	chrX:94,891,345	-		
		271-12L/SP6	chrX:94,854,496	+	131,966	140,000
		271-12L/T7	chrX:94,986,461	-		
*Tex13*	chrX:130,816,302	257-22B/SP6	chrX:130,761,904	+	124,211	120,000
		257-22B/T7	chrX:130,886,114	-		
		322-4J/SP6	chrX:130,761,839	+	117,691	110,000
		322-4J/T7	chrX:130,879,529	-		

BAC ends (BESs) of the positive clones identified on the membranes no. 6 and no. 7 were sequenced, mapped and aligned to the mouse genome (mm5 assembly, UCSC). The coordinates on the mouse genome are shown for the probes as well as for the ends of genomic inserts. An average insert size 140.8 kb was calculated from mapped positions and 141.2 kb was determined by pulsed-field gel electrophoresis (PFGE).

### Analysis of SNPs and DNA polymorphism

To find out the degree of nucleotide polymorphism between the PWD/Ph and C57BL/6J mouse strains, we aligned 73 uniquely mapped BESs (32,182 nucleotides) with their C57BL/6J genomic counterparts and found 297 single nucleotide substitutions. The calculated SNP rate 0.92 per 100 bp is significantly higher than SNP frequency between laboratory strains [18-20] and corresponds well to the rate between the closely related subspecies *Mus m. molossinus *and the C57BL/6J strain (0.96%) [21]. The insertions and deletions (indels) were found with lower frequency than SNPs: single nucleotide indels occurred with frequency 0.19% while multinucleotide indels with only 0.08% frequency. All nucleotide changes observed in the alignments of 87 PWD/Ph BESs and their B6 counterparts are summarized in Additional files 2 and 3. The high number of SNPs of the PWD/Ph strain is reflected by a high frequency of genetic and phenotypic variations between PWD/Ph and B6 inbred mice. An initial study performed to compare behavior of the PWD/Ph inbred strain with the B6 revealed substantial behavioral differences between these two strains [8]. Using dense SNP maps of various laboratory and wild-derived inbred strains [20,22] it will be possible to map genes responsible for particular complex traits more efficiently. For ultimate validation of candidate genes genomic BAC libraries will be highly desirable.

## Conclusion

The first genomic BAC library was constructed for the *Mus m. musculus *subspecies of the house mouse, represented by the PWD/Ph inbred strain. The quality of the PWD BAC library was verified by hybridization with seven unique probes that identified multiple positive clones. BAC end sequencing provided a new piece of evidence on the high incidence of SNPs (0.92/100 bp) between C57BL/6J and PWD/Ph inbred strains. The mouse PWD/Ph BAC library will serve as a tool for functional genomics of complex genetic traits with the ultimate goal to identify and clone responsible genes. The PWD BAC library will become accessible to the scientific community via RZPD, Berlin, Germany [23].

## Methods

### Mouse strain and DNA isolation

Mouse manipulation was in accordance with the Czech Animal Protection Act No. 246/92, 162/93, and decrees No. 311/97, fully compatible with the NIH Publication No. 85-23, revised 1985. A female mouse of the PWD/Ph inbred strain, derived from the *Mus mus musculus *subspecies [2] was used for high molecular weight DNA (HMW-DNA) preparation. The mouse was killed by cervical dislocation, spleen dissected and single cell suspension prepared in PBS using a glass homogenizer. The agarose-embeded HMW-DNA was prepared as described in detail elsewhere [24].

### Library construction

The agarose HMW-DNA plugs were subjected to pre-electrophoresis in a CHEF-DR-III apparatus (BioRad) in 1% agarose and 0.5 × TBE buffer for 12 hrs (4 V/cm, 5 s pulse, 14°C). Genomic DNA was partially digested with the mixture of *Eco*RI endonuclease and *Eco*RI methylase. The optimal ratio of the enzymes was determined by titration: usually 5–25 units of methylase per 1 unit of endonuclease were employed. DNA fragments were prepared by slight modification of an approach described before [24]. Briefly, DNA fragments were separated from the digested agarose plugs in the CHEF-DR-III in 1% agarose and 0.5 × TBE buffer for 16 hrs (5 V/cm, 0.1 to 40 s pulse, 14°C). Subsequently, three stripes corresponding to fragment size between 150 kb and 200 kb were excised and subjected to another size selection by additional electrophoresis in 0.5 × TBE buffer for 12 hr (5 V/cm, 2.5 to 4.5 s pulse, 14°C). The second size selection effectively removed short fragments while keeping long fragments in the agarose strips. The appropriate fragments were isolated by electroelution and ligated to the *Eco*RI site of the pBACe3.6 vector [25]. The ligation mixtures were dialyzed on ice in a well created by 0.5% agarose with 1 M glucose for 1 hr. The desalted ligation mixtures were electroporated into *E. coli *electrocompetent DH10B ElectroMax cells (Invitrogen) by a Gene Pulser apparatus (BioRad) in 0.1 cm cuvette with the following parameters: voltage 1.8 V, impedance 200 Ω, capacitance 25 μF, time constant between 3.5 to 4.5 s. The electroporated cells were diluted in 1 ml SOC medium and incubated in an orbital shaker at 37°C and 200 rpm for 1 hr. The titer of each electroporation reaction was determined by spreading an aliquot on selection agar plates (LB, 20 μg/ml chloramphenicol, 5% sucrose) as described [24]. The remainder containing the primary clones was supplemented with glycerol to the final concentration of 15%, then quickly frozen in liquid nitrogen and stored at -80°C. The frozen stocks of the primary clones were recovered and spread on large selection plates. The colonies were picked with multi-functional robotical system Gene TacTM-G3 (Genomic Solutions) and arrayed in 377 individual 384-well dishes containing LB medium supplied by 7.5% glycerol and 20 μg/ml chloramphenicol. The clones were gridded using the robot on 8 nylon membranes (18 342 unique clones in duplicates per membrane). Afterwards, the bacterial colonies were lysed, their DNA denatured, and crosslinked to the membranes by standard methods [26].

### Estimation of average insert size

One hundred sixty-two clones from the library segment 1 and 236 clones from the segment 2 were randomly picked and grown in 15 ml 2xYT medium (16 g/L tryptone, 10 g/L yeast extract, 5 g/L NaCl, 20 μg/ml chloramphenicol) at 37°C and 300 rpm for 18–20 h. BAC DNA was prepared using a modification of the standard protocol based on alkalic lysis. Briefly, 15 ml of the overnight culture was spun down, the bacterial pellet was resuspended in 300 μl of lysis buffer I (50 mM glucose, 25 mM Tris-HCl with pH = 8.0, 10 mM EDTA), then lysed with 600 μl of freshly made lysis buffer II (0.2 M NaOH, 1% SDS) and precipitated with 450 μl of lysis buffer III (3 M KOAc, pH = 4.8) followed by incubation on ice for 1 hour. The resulted precipitate was spun down in a microfuge for 15 min at maximum speed. The BAC DNA was further precipitated at room temperature with 0.6 volumes of isopropanol for 30 min and centrifuged at maximum speed for 10 min. The pellet was washed with 70% ethanol, air dried shortly and dissolved in 25 μl of TE. The BAC DNA was subjected to *Not*I (Fermentas) restriction overnight to achieve complete digestion. The reactions were resolved along with the mid range PFG marker I (New England Biolabs, cat # N3551S) in the CHEF-DR-III in 1% agarose and 0.5 × TBE buffer for 16 hrs (5 V/cm, 0.1 to 40 s pulse, 14°C). The insert size was estimated after ethidium bromide staining and the average insert size for both segments of the library was calculated.

### Hybridization of high-density colony filters

Seven probes for single-copy mouse genes were used to screen the library on high-density membranes. Six of them were produced by PCR on 50 ng of HMW-DNA isolated from the brain of a female PWD/Ph mouse. The primers were designed using Oligo 6 (MBI) software and the GenBank mRNA sequences (Table 1). PCR was performed at standard conditions: 96°C for 1 min, 30 cycles at 96°C for 10 s, 58°C for 20 s, 72°C for 1 min, and final extension at 72°C for 3 min. The 1085-bp DNA fragment of *Tbp *(U63933) was derived from the tbp-1942 cDNA clone (kind gift from Dr. Trachtulec) [27]. The resulted amplicons and *Tbp *fragment were purified and labeled by random priming with [α-^32^P]dCTP. Hybridization was done in Church buffer [28] with 15% formamide at 60°C over night. The membranes were washed in 0.2 × SSC, 0.1% SDS buffer at 42°C for 20 min and then in 0.2 × SSC, 0.1% SDS buffer at 60°C for 10 min. The membranes were then autoradiographed for 2 to 10 days, the positive clones identified and recovered from the frozen 384-well plates.

### BAC end sequencing and analysis

BAC DNA was prepared from 60 clones as described above and purified using a QIAGEN kit following the manufacturer's instructions. The sequencing was performed using a Big Dye Terminator v3.1 cycle sequencing kit in an ABI 310 instrument (Applied Biosystems) with primers T7 (GGTCGAGCTTGACATTGTAG) and SP6 (GATCCTCCCGAATTGACTAGTG). Each DNA sample was sequenced twice. The sequences from the same BAC end were aligned and manually edited in order to obtain a consensus sequence. BESs were masked for mouse repeats using RepeatMasker [29] (sensitive settings) and aligned to the mouse genome sequence (mm5 assembly, May 2004, UCSC) [30,31] using BLAT [32]. The mouse genome sequence had already been soft-masked for repeats by UCSC and BLAT was set to produce all possible alignments (tile size = 10, minimum score = 0, minimum sequence identity = 0). The hits were filtered to keep only those with minimum alignment ratio = 0.8. After manual inspection, a list of BESs mapped to unambiguous positions in the genome was compiled. The corresponding genomic sequences were excised and aligned with the appropriate BESs (unmasked sequence) using SSEARCH [33] (standard settings). A Perl script was written to process the pair-wise alignments and enumerate the sequence polymorphisms (SNPs, insertions, deletions, etc). The visualization of DNA polymorphisms was made by TeXshade LaTeX package [34]. All intermediate steps were performed using customized Perl scripts and utilities available from UCSC website [35].

## Authors' contributions

PJ constructed the library, participated in the sequence alignment and drafted the manuscript with JF and PD. PD carried out the sequence alignments and the analysis of DNA polymorphism. JF conceived the study, participated in its design and coordination. PJ and JF wrote the manuscript and all authors read and approved its final version.

## Supplementary Material

Additional file 1 BAC end sequences mapped on C57BL/6 mouse genome. Click here for file

Additional file 2 Summary of polymorphisms detected in 73 BAC end sequences. Click here for file

Additional file 3 Polymorphisms detected in 73 BAC end sequences. Click here for file
