# Supplementary material for: Construction and characterization of a genomic BAC library for the Mus m. musculus mouse subspecies (PWD/Ph inbred strain)
Source: BMC Genomics. 2005 Nov 16;6:161. doi: 10.1186/1471-2164-6-161 (PMC1299325; doi:10.1186/1471-2164-6-161)
Supplement: Additional file 3 — Polymorphisms detected in 73 BAC end sequences. [file 1471-2164-6-161-S3.pdf]

Additional file 3: Polymorphisms detected in 73 BAC end sequences.

```
>001-1A_T7 vs. chr2: 114,458,029-114,458,998 (+), 549 bp overlap

  1  TTAACCATCAGGGAAATGCAAATCAAAACAACCCTGAGAGTCCATCTCACACCAATCAGAATGGCTAAGA  001-1A_T7
     TTAACCATCAGGGAAATGCAAATCAAAACAACCCTGAGAGTCCATCTCACACCAATCAGAATGGCTAAGA  chr2

 71  TCAAAAATTCAGGTGACAGCAGATGCTGGCGAGGATGTAGAGAAAGATGAACACTCCTCCACTGTTGGTG  001-1A_T7
     TCAAAAATTCAGGTGACAGCAGATGCTGGCGAGGATGTAGAGAAAGATGAACACTCCTCCACTGTTGGTG  chr2

141  GGATTGCAAGCTTGTACAACCACGCTGGAAATCAGTCTGGGGATTTCTCAGAAAATTGGACATGATACTA  001-1A_T7
     GGATTGCAAGCTTGTACAACCACGCTGGAAATCAGTCTGGGGGTTTCTCAGAAAATTGGACATGATACTA  chr2

211  CCGGAGGATCCCGCAATACCTCTCCTGGGCATATATCCAGAAGATGTTCCAACTAGTAAGAAAGACACAT  001-1A_T7
     CCGGAGGATCCCGCAATACCTCTCCTGGGCATATATCCAGAAGATGTTCCAACTAGTAAGATAGACACAT  chr2

281  GCTCCACTATGTTCATAGCAGCCTTATTTATAATAGCCAGAAGCTGGAAAGAACCCAGATGCCTCTCAAC  001-1A_T7
     GCTCCACTATGTTCATAGCAGCATTATTTATAATAGCCAGAAGCTGGAAAGAACCCAGATGCCTCTCAAC  chr2

351  AGANNNNNGGATACCAAAC-TGTGGTACATTTACACAATGGAGTACTACTCAGCTATTAAAAAGAATGAA  001-1A_T7
     AGAGGAATGGATACAAAAAATGTGGTACATTTACACAATGGAGTACTACTCAGCTATTAAAAAGAATGAA  chr2

420  TTTATGAAATTCCTAGGCAAATGGTTGGACCTGGAGGGCATTATCCTGAGTGAGGTAACCCAATCACAA-  001-1A_T7
     TTTATGAAATTCCTAGGCAAATTGTTGGACCTGGAGGGCATTATCCTGAGTGAGGTAACCCAATCACAAA  chr2

489  GAACTCAAATGATATGTACTCACTGATAAGTGGATATTAGCCCAGAAACTTAGAATAACCA  001-1A_T7
     GAACTCAAATGATATGTACTCACTGATAAGTGGATATTAGCCCAGAAACTTAGAATACCCA  chr2
```

```
>001-1A_SP6 vs. chr2: 114,474,592-114,474,817 (-), 227 bp overlap

  1  GTCTCTCAAAACNACACAGGCAGGTAGACAGACAGGCAGACAAATGAAGTTGAGTGAATGGGTTCAAGTG  001-1A_SP6
     GTCTCTCAAAAC-ACACAGGCAGGTAGACAGACAGGCAGACAAATGAAGTTGAGTGAATGGGTTCAAGTG  chr2

 71  TTGTATCTCCAAATATTCTTAGTTTTAAGCAGATGTTGCTATTACCTATCACTTACCCTCCTCTTATACT  001-1A_SP6
     TTGTATCTCCAAATATTCTTAGTTTTAAGCAGATGTTGCTATTACCTATCACTTACCCTCCTCTTATACT  chr2

141  TATCATATCTTAAAGCTAATTGTCTATGCAGCTCAACTTCTCTGATAAGGACCATGCAGTTGAAGTTAGG  001-1A_SP6
     TATCATATCTTAAAGCTAATTGTCTATGCAGCTCAACGTCTCTGATAAGGACCATGCAGTTGAAGTTAGG  chr2

211  TCACTGCAGGTGAATTC  001-1A_SP6
     TCACTGCAGGTGAATTC  chr2
```

```
>008-2A_SP6 vs. chr1: 14,956,384-14,956,470 (+), 87 bp overlap

  1  GAATTCTAATAAGAATAGGAGAGAATCATCAGTCACTGAAAAACCTTTGAAGGTCCTGCCCGTGTGTGAC  008-2A_SP6
     GAATTCTAATAAGAATAGGAGAGAATCATCAGTCACTGAAAAACCTTTGAAGGTCCTGCCCGTGTGTGAC  chr1

 71  AGGGACCACAGAAGTTG  008-2A_SP6
     AGGGACCACAGAAGTTG  chr1
```

```
>016-3A_SP6 vs. chr19: 49,663,546-49,663,715 (-), 171 bp overlap

  1  TCCTCTTATTGCTCACCTTCAATGACTCCTTCTCTGTAAGCCTTCTCCTGCATACCTAGCTTCTCTCTGG  016-3A_SP6
     TCCTCT-ATTGCTCACCTCCAATGACTCCTTCTCTGTAAGCCTTCTCCTGCATACCTAGCTTCTCTCTGG  chr19

 71  CCTTATTCTCCAAAGGCTTGTGGTCCAACAGTGCAGTCCTTCCAACTACTGATCACATGTATATATATAG  016-3A_SP6
     CCTTATTCTCCAAAGGCTTGTGGTCCAACAGTGCAGTCCTTCCAACTACTGATTACATGTATATATTTAG  chr19

141  TTGTAGTCTCCATAAATGCTTGATATCAAAG  016-3A_SP6
     TTGTAGTCTCCATAAATGCTTGATATCAAAG  chr19
```

>016-3A_T7 vs. chr19: 49,530,675-49,530,901 (+), 227 bp overlap

```
  1  TTTTTTGTCCATTGTCAGAACATCAGAATAAAGAAAATGTCCAGGTGTCATTGTGGGTCACAATGAATTG  016-3A_T7
     TTTTTTGTCCATTGTCAGAACATCAGAATAAAGAAAATGTCCAGGTGTCATTGTGGGTAACAATGAATTG  chr19

 71  CAATCGTTACTGAACTGTTTGGCCAAGCTCAGTAGTTTTGAGTGCTGCCAGCCACCGGGAATGATCCTAG  016-3A_T7
     CAATCGTTACTGAACTGTTTGGCCAAGCTCAGTAGTTTTGAGTGCTGCCAGCCACCAGGAATGATCCTAG  chr19

141  GTGCTCAGAACACCTGACACAGGGTGGAGCTTCTAGTTTTGAAGAGGGAAGGCAGTTTTGAAGAGGGAAG  016-3A_T7
     GTGCTCAGAACACCTGACACAGGGTGGAGCTTCTAGTTTTGAAGAGGGAAGGCAGTTTTGAAGAGGGAAG  chr19

211  GCAGTGAAGTACGAAGC  016-3A_T7
     GCAGTGAAGTACGAAGC  chr19
```

>032-11C_SP6 vs. chr9: 5,307,663-5,307,872 (-), 210 bp overlap

```
  1  CAAAATTCCAACTCAATTCTTCATAGAGCTACAAAGAGCAATTTGCAAATTCATTTGGTATAACAAAAAA  032-11C_SP6
     CAAAATTCCAACTCAATTCTTCATAGAGCTACAAAGAGCAATTTGCAAATTCATTTGGTATAACAAAAAA  chr9

 71  TGAAAGATAGCAAAAGCTATTCTCAAAAATACAACAACTTCCTTCTCCTTGATTTTCTTGTGTTTTGCAA  032-11C_SP6
     TGAAAGATAGCAAAAGCTATTCTCAAAAATACAACAACTTCCTTCTCCTTGATTTTCTTGTGTTTTGCAA  chr9

141  ATTGTATTTTGGGTGTTCTATGTTTGTGGGCTAATACCCACTTATCAGTGAGTGCACATCTAATGAATTC  032-11C_SP6
     ATTGTATCTTGGGTGTTCTATGTTTCTGGGCTAATACCCACTTATCAGTGAGTGCACATCTAATGAATTC  chr9
```

>032-11C_T7 vs. chr9: 5,196,339-5,196,861 (+), 527 bp overlap

```
  1  TCTCAAATACTTAAAAAAATTGTCCCATGAAATATGACTGTGTGGATCTGGGGGTGTAGGGGAAGGTGGG  032-11C_T7
     TCTCAAATACTTAAAAAAATTGTCCCATGAAATATGACTGTGTGGATCTGGGGGTGTAGGGGAAGGCGGG  chr9

 71  GGTAGAGCCCACATCTCCTCAGAATTCCTGTGCTCTGGGCAGGCAGAATCAGAAGGACTGCCAGATGCTT  032-11C_T7
     GGTAGAGCCCACATCTCGTCAGAATTCCTGTGCTCTGGGCAGGCAGAATCAGAAGGACTGCCAGATGCTT  chr9

141  TCCACTTGGCCCCAGGTGGGAATCTGGCTGTGTGAAGCCACTGACCTCACTCAGTGGGGCGTGGACAAGG  032-11C_T7
     TCCACTTGACCCCAGGTGGGAATCTGGCTGTGTGAAGCCACTGACCTCACTCAGTGAGGCGTGGACAAGG  chr9

211  GGTAGCCCCTGTACCAGGTTCTCAGTCTCCGGCATCCCATGCTGGATACCGCAGAGAATCTGAGAACAGA  032-11C_T7
     GGTAGCCCCTGTACCAGGTTCTCAGTCTCCGGCATCCCATGCTGGATACGGCAGAGGATCTGAGAACAGA  chr9

281  ATAGGGGATACAGGAAGAGGGAAGAGGGAGAGATGTTTCCACACAGGTGAGAGTCCTTGGTCCGGGCCTT  032-11C_T7
     ATAGGGGATACAGGAAGAGGGAAGAGGGAGAGATGTTTCCACACAGGTGAGAGTCCTTGGTCCGGGCCTT  chr9

351  GACTGGAGCACAGGAAGGCCTTCTGATGAGAGATTAGGCACAGCTCCTTAGGAAAAAGTCTATCCCATTG  032-11C_T7
     GACTGGAGCACAGGAAGGCCTTCTGATGAGAGATTAGGCACAG----TTAGGAGAAAGTCTATCCCATTG  chr9

421  TCCAAGTACAGCAGGCCTTGATGAACAGAGACAGTCTAGAGTTTTAGAGCTTTATTGTAGAAAGGCAGGA  032-11C_T7
     TCCAAGCACAGCAGGCCTTGATGAACAGAGACAGTCTAGAGTTTTAGAGCTTTATTGTAGAAAGGCAGGG  chr9

491  TGAAAGAGAAAAGGTAGAAAGAGAGAAACCTGCCATG  032-11C_T7
     TGAAAGAGAAAAGGTAGAAAGAGAGAAACCTGCTATG  chr9
```

>048-7A_SP6 vs. chr9: 32,904,919-32,905,421 (+), 504 bp overlap

```
  1  GAATTCCTTAAAGATAAGGCTCAGTATACAATCTTCTTGAGATCACACTGATGGGTTATGTGTAATACTT  048-7A_SP6
     GAATTCCTCAAAGATAAGGCTCAGTATACAATCTTCTTGAGATCACACTGATGGGTTATGTGTAATACCT  chr9

 71  CCAGACTAAATTTATGGGTGTCACTAGGCTGTGGATAAATTTGTTCAGATGGAATTCTTTGATTAGATCC  048-7A_SP6
     CCAGACTAAATTTATGGGTGTCACTAGGCTGTGGATAAATTTGTTCAGATGGAATTCTTTGATTAGATCC  chr9
```

```
141   ATTTTTGAAAAATGATAAAACCCGTCAGAAAAATGTTTTCACTCTCAGACTTGTAAAGTGGAAATTGCCC   048-7A_SP6
      ACTTTTGAAAAATGATAAAACCCATCAGAAAAATGTATTCACTCTCAGACT-GTAAAGTGGAAATTGCCC   chr9

211   CTCTAAAGAAGCCACATGGTAGCCAGACCTGATATCACAGGCCTCCCTTTCCAACCAATCTGGACGCTGG   048-7A_SP6
      CTCTAAAGAAGCCACATGGTAGCCAGACCTGGTATCACAGGCCTCCCTTTCCAACCAATCTGGACGCTGG   chr9

281   AGCAGGAGGATAGCAAGTTTAAAGCCTGCCTAGGTTACAAAGTGAATTCAGGAACAGCCTCAGCAATATA   048-7A_SP6
      AGCAGGAGGATAGCAAGTTCAAAGCCTGCCTAGGTTACAAAGTGAATTCAGGAACAGCCTCAGCAATATA   chr9

351   ATGCGACTTCATCTTGAAACAAAACGTTAAGAGATGGCTGGGAGTGTGCCTCAATGATAAAAATGTACCT   048-7A_SP6
      ATGCGACCTCATCTTGAAACAAAACGTTAAGAGATGGCTGGGAGTGTGCCTCAATGATAAAAATGTACCT   chr9

421   TACATTCACAGAGCCCTAGGCTCCATCTCTAATACAGTGAGAAAAAATAAAGAAGGCAAAAAATGTTATA   048-7A_SP6
      AACATTCACAGAGCCCTAGGCTCCATCTCTAATACAGTGAGAAAAAATAAATAAGGCAAAAAATGTTATA   chr9

491   TGTGCTATTTCAGG   048-7A_SP6
      TGTGCTATTTCAGG   chr9
```

```
>048-7A_T7 vs. chr9: 33,012,575-33,012,986 (-), 412 bp overlap
```

```
  1   TTGCTGATACGGCGGTTTGTATGATTGTTTCTGAATGCTGAATTAGTCCTGCATGTGTGAAAGAAAGCCC   048-7A_T7
      TTGCTGATACGGCGGTTTGTATGATTGTTTCTGAATGCTGAATTAGTCCTGCATGTGTGAAAGAAAGCCC   chr9

 71   AGTTAGTCATGGGGTGGAATTCATTTTGTACATTGCTGTACTCAATTTGCTAGATTTTATTGTAGGCATT   048-7A_T7
      AGTTAGTCATGGGGTGGAATTCATTTTGTACATTGCTGTACTCAATTTGCTAAATTTTATTGTAGGCATT   chr9

141   TACATCTAAATTAGTGAGGGATATTCATCTATTATTTTTCTGGTACTTTCTTTTCTTCTTTTCTGGCTTT   048-7A_T7
      TACATCTAAATTAGTGAGGGATATTCATCTATTATTTTTCTGGTACTTTCTTTTCTTCTTTTCTGGCTTT   chr9

211   GGTATCAGGACAATTTTAATCTCATGAAAATCACACAGAGAGAGGCTATATAAGATTGATTTGAATTTTT   048-7A_T7
      GGTATCAGGACAATTTTAATCTCATGAAAATCACACAGAGAGAGGCTATATAAGATTGATTTGAATTTTT   chr9

281   TTTTAATGCTTAATAGAATTCTTGAGTGAAACTACATGGGCCAATATNNATCCTTTTTGTAAGATTTTCA   048-7A_T7
      TTTTAATGCTTAATAGAATTCTTGAGTGAAACTACATGGGCCAATATACATCCTTTTTGTAAGATTTTCA   chr9

351   TTACAAATTTGATTTCTTGATAGGCACAGTACTATTCAGGTGAATGGTTTCGTGTTAAGGAG   048-7A_T7
      TTACAAATTTGATTTCTTGATAGGCACAGTACTATTCAGGTGAATGGTTTCGTGTTAAGGAG   chr9
```

```
>072-10A_SP6 vs. chr6: 93,249,869-93,250,399 (-), 533 bp overlap
```

```
  1   TTCTAAAATTTAATCTATTTTTTAATTTAAAAGCAAAATAGAAGTACCCAGAAAGAATAATTGTCTGTCT   072-10A_SP6
      TTCTAAAATTTAACCTATTTTTTAATTTAAAAGCAAAATAGAAGTACCCAGAAAGAATAATTGTCTGTCT   chr6

 71   TTCAAAACTGGGTTTTTGTTCTGATTTGTTTCTCTTTCCTATTTCCCTATTTCCTATTTGTTTTCCTTTT   072-10A_SP6
      TTCAAAACTGGGTTTTTGTTCTGATTTGTTTCTCGTTCCTATTTCCCTATTTCCTATTTGTTTTCCTTTT   chr6

141   TCTATTTCCTATTTTCCTGTTTCCTATCAACCGGTTCTGGATAGGTTCTTTTTCCTGCCATCAGGAATCC   072-10A_SP6
      TCTATTTCCTATTTTCCTGTTTCCTATCAACCGGTTCTGGATAGGTTCTTTTTCCTGCCATCAGGAATCC   chr6

211   CTGAGCCAAATTTGGGAAGAAAACAACAAGATAAGGACCCTCAAGTCTGGTGGTGCCCCTTCTTGCCCTA   072-10A_SP6
      CTGAGCCAAATTTGGGAAGAAAACAACAAGATAAGGACCCTCAAGTCTGGTGGTGCCCCTTCTTGCCCTA   chr6

281   GTTAGTGCTGGATGCGCCAACTTTTAATCTCTCTGTTCACTCTGTGCATGCTATCCCAAACTCTAGGGTG   072-10A_SP6
      GTTAGTGCTGGATGCGCCAACTTGTAATCTC--TGCTTACTCTGCGCATGCTATCCCAAACTCTAGGGTG   chr6

351   CAATGACATAGCCTGATCATTAAGAGCAGCCAACGCAAAAGCATCAATTGTCAGACAAACTTAAAGAATA   072-10A_SP6
      CAATGACATAGCCTGATCATTAAGAGCAGCCAACGCAAAAGCATCAATTGTCAGACAAACTTAAAGAATA   chr6

421   ACAGCAAATCTGGAATGGAAAAGAATTCCATGGAATTAAAGTGCCTTGGGTACCTTGGGTCTCCCAGGCC   072-10A_SP6
      ACAGCAAATCTGGAATGGAAAAGAATTCCATGGAATTAAAGTGCCTTGGGTACCTTGGGTATCCCAGGCC   chr6

491   TGAAGTCCACCAGCTCTCTCCACAGCCCCCAGCTGAAGAATTC   072-10A_SP6
      TGAAGTCCACCAGCTCTCTCCACAGCCTCCAGCTGAAGAATTC   chr6
```

```
>072-10A_T7 vs. chr6: 93,125,121-93,125,326 (+), 207 bp overlap

  1  AGTTTTAAAGTCAAGTAGAGGAGCATTCCGGGGGGTAGGTGATCTATATCTTGATTGATGGGTTCTATCT  072-10A_T7
     AGTTTTAAAGTCAAGTAGAGGAGCATTCCGGGGGGTAGGTGATCTATATCTTGATTGATGGGTTCTATCT  chr6

 71  CTGGGAGTCTGGATGATGCTATGATTCAGACTAATTATTCTTGTTTCATGCCAAGGAAGGGTAGTTCTTG  072-10A_T7
     CTGGGAGTCTGGATGATGCTATGATTCAGACTAATTATTCTTGTTTCATGCCAAGGAAGGGTAGTTCTTG  chr6

141  ACCTCTTGGCCACAACCTTGGCTAACTGTCCTTCGTAAGCCTGAGGTTTGTTGCAGTAACTGACTTA  072-10A_T7
     ACCTCTTGGCCACAACCTTGGCTAACTGTCCTTCGTAAGCCTGAGGTT-GTTGCAGTAACTGACTTA  chr6
```

```
>088-12A_SP6 vs. chrX: 141,997,504-141,997,948 (-), 460 bp overlap

  1  TTTTTCCAGGCTTCTGGGCTATCACAAATAAGACTGCTATGAACACAGTGGAACACGTGCCCCTGTGACA  088-12A_SP6
     TTTTTCCAG-CTTCTGG-CTATCACAAATAAGACTGCTATGAACACAGTGGAACACGTGCCCCTGTGACA  chrX

 71  TGGTTGGGGTGGGGCATCTTTTGGATATATATATATATATATATATATATATATATATATATACATATAT  088-12A_SP6
     TGGT-GGGGTGGGGCATCTTTTGGATATATATATATATATATATATATATATATATATA---------  chrX

141  ATATATAATCTTTTCTCCATTAAATTACCTTTGCTCCCTTGTTCAAGATCATTGATTAGATCTGTGCTTA  088-12A_SP6
     ---TATAATCTTTTCTCCATTAAATTACCTTTGCTCCCTTGTTCAAGATCATTGATTAGATCTGTGCTTA  chrX

211  CTTCCATCTTCTCTGATTTTTTTGTTATGGAAAAAGGGTTTATTTTGAAATTATTTTAATTTAGAAATAG  088-12A_SP6
     CTTCCATCTTCTCTGATTTTTTTGTTATGGAAAAAGGGTTTATTTTGAAATTATTTTAATTTAGAAATAG  chrX

281  AAAACCTCATAGGAAGGAGGAAAGAAACAAAATACATAAAGCATCCAGGCAACGTTTTACTTCATGAGCT  088-12A_SP6
     AAAACCTCATAGGAAGGAGGAAAGAAACAAAATACATAAAGCATCCAGGCAACGTTTTACTTCATGAGCT  chrX

351  CATCACTATCTCATTGGTTCACTTGTCCCATGATGAGAAAGGACTCAGCCACCCATAGTAATGTTATTAA  088-12A_SP6
     CATCACTATCTCATTGGTTCACTTGTCCCATGATGAGAAAGGACTCAGCCACCCATAGTAATGTTATTAA  chrX

421  TAGGGATATGGATCAGTTTCACAAAGAAGCCAATGAATTC  088-12A_SP6
     TAGGGATATGGATCAGTTTCACAAAGAAGCCAATGAATTC  chrX
```

```
>096-1B_T7 vs. chr4: 29,734,830-29,735,232 (+), 415 bp overlap

  1  CTGTACTTCAGTTTGCTTTTAAATTGTATGTGACTAATATTAATAAATATAAAAATACAATTTGTGGTTA  096-1B_T7
     CTGTACTTCAGTTTGCTTTTAAATTGTATGTGACTAATATTAATAAATATAAAAATACAATTTGTGGTTA  chr4

 71  TTCAATGTTTTTGTTTTCTATATATATTTTAAGGCTGACCACTTGGTATCAGATAAAGTGTTAGGGGCTC  096-1B_T7
     TTCAATGTTTTTGTTTTCTATATATATTTTAAGGCTGAACACTTGGTATCAGATAAAGCGTTAGGGGCTC  chr4

141  ATCCCTGGGGAAGTCTGATTCTCCCTCTCTAGGTATTAATTGTATGTAATTCTTAATCTGGGGATGGGGT  096-1B_T7
     ATCCCTGGGGAAGTCTGATTCTCCCTCTCTAGGTATTAATTGTATGTA----------TGGGGATGGGGT  chr4

211  CAAGNGAGATTTTCTTTACCCTCTTTGAGACGTCAGTTTATGGTGTCATTATCCAGGCCTTGTTTAGCCG  096-1B_T7
     CTTGTGAGATTTTCTTTACCCTCTTTGAGACGTCAGTTTATGGTGTCATTATCCAGGCCTTGTTTAGCCA  chr4

281  ACAGTATTGTTTAGAGTTTACA-GTGAGATAGCCTATCAAATATAAAACATACAATCTTGTAACAGACTT  096-1B_T7
     ACTATATTGTTTAGAGTTTACAAGTGAGATAGCCTATCAAATATAAAACATACAATCTTGTAACAGACTT  chr4

350  CATGAATCTTCTGGCTGTTAACAAATTATTCTATCCCTGTTCCCTGGGTGCCTTTGTCCTTATGTG  096-1B_T7
     CATGA-TCTTCTGGCTGTTAACAA-TTATTCTATCCCTGTTCC-TGAGTGCCTTTGTCCTTATGTG  chr4
```

```
>120-4B_SP6 vs. chr15: 40,526,985-40,527,497 (-), 513 bp overlap

  1  TTTACAGGTTGAGTAAGAATTTTCTTTACACATGAAAGACATATTGTAGAGTCTTTGTAGCGAAAAGATT  120-4B_SP6
     TTTACAGGTTGAGTAAGAATTTTCTTTACACATGAAAGACATATTGTAGAGTCTTTGTAGCGAAAAGATT  chr15
```

```
 71   CTGCAGTTGAAAAGGTTGAGATAAATTGTCTAACTAATGCCTGCCAATGGTCAGACTCCAGTGTCTTCAA   120-4B_SP6
      CTGCAGTTGAAAAGGTTGAGATAAATGGTCTAACTAATGCCTGCCAATGGTCAGACTCCAGTGTCTTCAA   chr15

141   AAGACAAGGGCAGGGATCATCGCAAGAAAGATAATTTTCTATTTAAATTTTGGGCTCCCAAACTAACATT   120-4B_SP6
      AAGACAAGGGCAGGGATCATCGCAAGAAAGATAATTTTCTATTTAAATTTTGGGCTCCCAAACTAACATT   chr15

211   TTGCTAATTATGACAATGGAGTCTGCTCCTAGCACCAAAGGACCACCCCTTTCACCCGTCCTTTGAAACT   120-4B_SP6
      TTGCTAATTATGACAATGGAGTCTGCTCCTAGCACCAAAGGACCACCCCTTTCACCCGTCCTTTGAAACT   chr15

281   ACTATAACCCGCCACCTGGTATGTTCTTTTTATATCCACTATAAACTACAGCTTTAATAAATATTTATTG   120-4B_SP6
      ACTATAACCCGCCACCTGGTATGTTCTTTTTATATCCACTATAAACTACAGCTTTAATAAATATTTATTG   chr15

351   GGGGAAACTAAAGAATCCTGATATGGAGAAGGGACCAGTGAAAACAAAGCTTTCTTCACTCTCAGTATTT   120-4B_SP6
      GGGGGAACTAAAGAATCCTGATATGGAGAAGGGACCAGTGAAAACAAAGCTTTCTTCACTCTCAGTATTT   chr15

421   CTCAAGCTTTTTTTGGTGGGGTGGGGGGTGGGAGGGAGGCAGAGCTCAAGCCTTGTTAGTACCCTGTGCT   120-4B_SP6
      CTCAAGCTTTTTTTGGTGGGGTGGGGGGTGGGAGGGAGGCAGAGCTCAAGCCTTGTTAGTACCCTGTGCT   chr15

491   GTCCATTTCCCCTTACTGAATTC   120-4B_SP6
      GTCCATTTCCCCTTACTGAATTC   chr15
```

>120-4B_T7 vs. chr15: 40,396,461-40,396,493 (+), 33 bp overlap

```
  1   CTCCCCATCCTTGCCTGGAATTTTGGTTGGCCT   120-4B_T7
      CTCCCCATCCTTGCCTGGAATTTTGGTTGGCCT   chr15
```

>128-5_SP6 vs. chr7: 59,269,790-59,270,000 (+), 211 bp overlap

```
  1   ATGAAAAACTATGCTAGGGTAAATTCAAGTCAGTGGTGAAGATGGGGGCAGCAAACACAGGAGGCAGTCC   128-5_SP6
      ATGAAAAACTATGCTAGGGTAAATTCAAGTCAGTGGTGAAGATGGGGGCAGCAAACACAGGAGGCAGTCC   chr7

 71   AGACTCACTTTCCTTGGTCCTTCATGAGCACTGAGAAAGGATTATGTGGAAACTGGCTCATCAGAGTGGT   128-5_SP6
      AGACTCACTTTCCTTGGTCCTTCATGAGCACTGAGAAAGGATTATGTGGAAACTGGCTCATCAGAGTGGT   chr7

141   GAGCATCATACATCCCAAGCATGGCAGAAGCATGCCAGACCCTCAAAACAAAACTGAGCACCNGCATCCC   128-5_SP6
      GAGCATCATACATCCCAAGCATGGCAGAAGCATGCCAGACCCTCAAAACAAAACTGAGCACCAGCATCCC   chr7

211   C   128-5_SP6
      C   chr7
```

>128-5B_T7 vs. chr7: 59,381,337-59,381,546 (-), 186 bp overlap

```
  1   TTTTGGCTCAGCCTGCAATGAAACACTCCCATCACTGGATGTCTATAGGGAGGAAAAGTATATCTAGGGT   128-5B_T7
      TTTTGGCTCAGCCTGCAATGAAACACTCCCATCACTGGATGTCTATAGGGAGGAAAAGTATATCTAGGGT   chr7

 71   GAGCAGTAGAGTCTGTCAAGAAACACACCCACAGCCATGAGATCTCAGTGCAAGCGATA---AACAAGAG   128-5B_T7
      GAGCAGTAGAGTCTGTCAA-AAACACACCCACAGTCATGAGATCTCAGTGCAAGCAAAACAAAACAAAAC   chr7

138   ACAA--AGACAAAACGNCAACAACAACAACAACAACAACAAACAGATCTAC   128-5B_T7
      AAAACAAAACAAAACAACAACAACAACAACAACAACAACAACAACAACAAC   chr7
```

>136-6B_SP6 vs. chr13: 17,084,203-17,084,861 (+), 647 bp overlap

```
  1   GAATTCTGTATGTACTCTTGGAATACCAAAAGGGTCTCTAAAGACAAACCACAAGGAAAAGATTATTAAC   136-6B_SP6
      GAATTCTGTATGTACTCTTGGAATACCAAAAGGGTCTCTAAAGACAAACCACAAGGAAAAGATTATTAAC   chr13

 71   TACAGAAAACATTCAAATTTTTGTATAACAAGACATTCAAAGGCAAGTACTTTGGAAGACACATAGGTCA   136-6B_SP6
      TACAGAAAACATTCAAATTTTTGTATAACAAGACATTCAAAGGCAAGTACTTTGGAAGACACATAGGTCA   chr13
```

```
141   GCAAGGGCTACCTTCAAAACCACAGAAAAAAACTTTAGACAAGTCAGTCTACCAAATAAAGTAATGAAAT   136-6B_SP6
      GCAAGGGCTACCTTCAAAACCACAGAAAAAAACTTTAGACAAGTCAGTCTACCAAATAAAGTAATGAAAT   chr13

211   TCACTAGTAATTAGAGAAACACAAATTTAAAA-GATTGTCACCTTACAAACATAAAAAACAA---GCCCC   136-6B_SP6
      TCACTAGTAATTAGAGAAACACAAATTTAAAAAGATTGTCACCTTACAAACATAAAAAACAAAAAGCCCC   chr13

277   TTCTGTGTTCCTACTCCTAAAGACAATTCTATGAAAGGGGAAAGTGTGAAGTTCTAAGCTTTGTCCAACA   136-6B_SP6
      TTCTGTGTTCCTACTCCTAAAGACAATTCTATGAAAGGGGAAAGTGTGAAGTTCTAAGCTTTGTCCAACA   chr13

347   ACAAATAGCCCTCCCCATCACCACCCAAAAAAAAACACATGGCACAGTAAATCATGAGTGGCCTTTTCTA   136-6B_SP6
      ACAAATAGCCCTCCCCATCACCACCCAAAAAATAACACATGGCACAGTAAATCATGAGTGGCCTTTTCTA   chr13

417   AAGAGTCTGCTTTGAATATACACAAAAAGAAA-TACAACTTTAAAGATGCCCTGGGATTCTTACAGAAGT   136-6B_SP6
      AAGAGTCTGCTTTGAATATACACAAAAAGAAAATACAACTTTAAAGATGCCCTGGGATTCTTACAGAAGT   chr13

486   TTATTTATTGACTATCAA-TACAACTTTTCTTTTTCCCTCTTAAAAACAAATGGTCTTTACATTAA-CTG   136-6B_SP6
      TTATTTATTGACTATCAAATACAACTTTTCTTTTTCCCTCTTAAAAACAAATGGTCTTTACATTAAACTG   chr13

554   A-GTATAATAACATTTTAAAATTCTTGCAAAAGAATACCTTGGG-CTGGTGAGATGGCTTA-CGGGTAAG   136-6B_SP6
      AAGTATAATAACATTTTAAAATTCTTGCAAAAGAATACCTTGGGGCTGGTGAGATGGCTTAGCGGGTAAG   chr13

621   AACACTA-CTGCTCTTCGGA-GGTCCTGA   136-6B_SP6
      AACACTAACTGCTCTTCCGAAGGTCCTGA   chr13
```

```
>144-7B_SP6 vs. chr19: 3,257,612-3,257,787 (-), 176 bp overlap

  1   ATCTCCTACCCAGATCTCCTACTCCCTTGGGGGGGTGGGGGGGTGAGGTGGGGGCATAAGCAAGAGGGCA   144-7B_SP6
      ATCTCCTACCCAGATCTCCTACTCCCTTGGGTGGGTGGGGGGGTGAGGTGGGGGCATAAGCAAGAGGGCA   chr19

 71   CAAATGTGGACCAGCTGAACAAGCCTAAGGTTTGGAGTTTCACAAGTAGACTGTAAAGAAATGTTTAAAA   144-7B_SP6
      CAAATGTGGACCAGCTGAACAAGCCTAAGGTTTGGAGTTTCACAAGTAGACTGTAAAGAAATGTTTAAAA   chr19

141   TTGCCTGAGCCTAATTTGATTCTCCTAGTGAATTCC   144-7B_SP6
      TTGCCTGAGCCTAATTTGATTCTCCTAGTGAATTCC   chr19
```

```
>144-7B_T7 vs. chr19: 3,132,629-3,132,986 (+), 359 bp overlap

  1   TATTCACAGAGCACGTGCCACTGTGTAGCATTGTAACCAGTTAGGGAACATTTTTATCAACCCTCCCAAA   144-7B_T7
      TATTCACAGCA-ACGTGCCACTGTGTAGCATTGTAACCAGTTAGGGAACATTTTTATCAACCCTCCCAAA   chr19

 71   TACTCCTTCTTAGCCTCATCCAGCCTCCCCTGGTGTGTCCCTGGCTGCTCTGGTCTTTCCTTCTCTGTAG   144-7B_T7
      TACTCCTTCTTAGCCTCATCCAGCCTCCCCTGGTGTGTCCCTGGCTGCTCTGGTCTTTCCTTCTCTGTAG   chr19

141   GTTTGTTCTGTTCTGGACACGTCATGTAAATGGAGTGAGACATTGGCTTCCTTGGCTTCCTGCCATGCTG   144-7B_T7
      GTTTGTTCTGTTCTGGACACGTCATGTAAATGGAGTGAGACATTGGCTTCCTTGGCTTCCTGCCATGCTG   chr19

211   GTTTGGTTTGTCTTGGTTTTGTGAGACAGGGTCTCCTGTAGCTCAGGCTAACTTCTAGCTAANNNNGATT   144-7B_T7
      GTTTGGTTTGTCTTGGTTTTGTGAGACAGGGTCTCCTGTAGCTCAGGCTAACTTCTAGCTAAGGATGATT   chr19

281   TGAACATCTGATCCTCCCACCTCCTCTCTCAAGTGCTCTGACCTCAGGAGAGTGCCACCATGCTGTGTTC   144-7B_T7
      TGAACATCTGATCCTCCCACCTCCTCTCTCAAGTGCTCTGACCTCAGGAGAGTGCCACCATGCTGTGTTC   chr19

351   ATGTGTCGC   144-7B_T7
      ATGTGTCGC   chr19
```

```
>152-7B_T7 vs. chr2: 180,150,561-180,150,943 (-), 392 bp overlap

  1   CCGGGAATTCCAACTTTAGAAAAACAAAATCAACAACAAGAAAGCCATGTGTTCCAGGGCACAGAATTCA   152-7B_T7
      CCGTGAAATCCAACTTTAGAAAAACAAAATCAACAACAAGAAAGCCATGTGTTCCAGGGCACAGAATTCA   chr2
```

```
 71  GGCATTACTCCTTGCAAGGCAAACACTTTTACCAGCTGAGCTCCGGTGTCGCTCTCAAACATTTATGTTT  152-7B_T7
     GGCATTACTCCTTGCAAGGCAAACACTTTTACCAGCTGAGCTCCGGTGTCGCTCTCAAACATTTATGTTT  chr2

141  TATTTATGTGNCTGAGTGGAGTTTATGCCTTCTATGTGTGGGTGCCCACAGAGGACAGAAAAGGGCATTT  152-7B_T7
     TATTTATGTGGCTGAGTGGAGTTTATGCCTTCTATGTGTGGGTGCCCACAGAGGACAGAAAAGGGCATTT  chr2

211  GATCTCCTGGAGCTGAAGTTATAGGTAGTTGTAAGACACCTTCAGGTGCTGGCAACAGAACTCGGGTCCT  152-7B_T7
     GATCTCCTGGAGCTGAAGTTATAGGTAGTTGTAAGACACCTTCGGGTGCTGGGAACAGAACTCGAGTCCT  chr2

281  CTGGGAGATACCGCCTTAACTGCTGAGTCATCACTACAGCCTTACAGGCCCCCCCCTTTTTTTTTTTTGA  152-7B_T7
     CTGGGAGATACCGCCTTAACTGCTGAGTCATCACTACAGCCTTACCGGCCCCTTT-TTTTTTTCTTTTGA  chr2

351  GTCAGAATCTCCAGCTTAGATTGGTCTTGAACTTGTAATCCT  152-7B_T7
     GTCAGAATCTCCAGCTTAGATTGGTCTTGAACTTGTAATCCT  chr2
```

>160-9B_SP6 vs. chr14: 79,957,422-79,957,995 (+), 573 bp overlap

```
  1  TCTTTATAAAATAGCAATTCACATGTCACAAAAACTATATTCCCCTATTGTATGAGAAGGGAACTTTTTA  160-9B_SP6
     TCTTTATAAAATAGCAATTCACATGTCACAAAAACTATACTCCCCTATTGTATGAGAAGGGAACTTTTTA  chr14

 71  TTTTGTCTGTTTACTATAATTTCAGAGGAGATTTGCTAGACCAAAAGGAAATCTTCTAAATTATGCTAAG  160-9B_SP6
     TTTTGTCTGTTTACTATAATTTCAGAGGAGATTTGCTAGACCAAAAGGAAATCTTCTAAATTATGCTAAG  chr14

141  TAGACAGCCTTGAATACAATCAAATGGGTAGTGAGTGACTCTGCAAGAGGAAAAATTTGAGGACTAACTA  160-9B_SP6
     TAGACAGCCTTGAATACAATCAAATGGGTAGTGAGTGACTCTGCAAGAGGAAAAATTTGAGGACTAACTA  chr14

211  CCATGAATCCCATATAAGCAAAAGGAAATATTAAGTGTCAGAAAAAGGAAAGGAACAGGCCAATAGGATT  160-9B_SP6
     CCATGAATCCCATATAAGCAAAAGGAAATATTAAGTGTCAGAAAAAGGAAAGGAACAGGCCAATAGGATT  chr14

281  CTGCATGAAGTGGGTCAGCTGTAACATTGAAGGCAAGTGACTCAGGGATGTAAAAATGACAAGCTTTAGT  160-9B_SP6
     CTGCATGAAGTGGGTCAGCTGTAACATTGAAGGCAAGTGACTCAGGGATGTAAAAATGACAAGCTTTAGT  chr14

351  GGATTCCACTGAGAATTCAAGAAGGAAGAGGGTTGGNAAGCACCCAATATCCTAGCAAGGAATATCAGTT  160-9B_SP6
     GGATTCCACTGAGAATTCAAGAAGGAAGAGGGCTGGGAAGCACCCAATATCCTAGCAAGGAATATCAGTT  chr14

421  AGTATAATGGATAAGAGTTGTAGTAGATTAATAAGTACTTTAGGGCAGTTTAAAGACAA-GCTAGAAGAC  160-9B_SP6
     AGTATAATGGATAAGAGTTGTAGTAGATTAATAAGTACTTTAGGGCAGTTTAAAGACAAAGCTAGAAGAC  chr14

490  ACGAAATAGAGAGTAGACAAACAGACTAATTATTGGCAGAATAATAGAGGAAGGCGAGATTTTTTCATAT  160-9B_SP6
     ACGAAATAGAGAGTAGACAAACAGACTAATTATTGGCAGAATAATAGAGGAAGGCGAGATTTTTTCATAT  chr14

560  GCATATTTATGAAT  160-9B_SP6
     GCATATTTATGAAT  chr14
```

>160-9B_T7 vs. chr14: 80,075,786-80,076,150 (-), 365 bp overlap

```
  1  CCCTACAGTGGGACATCAAGCCTTCAGAAGACCAAGGGCCTTTCCTCCCATTGATGTCCTACAAGGCCAT  160-9B_T7
     CCCTACAGTGGGACATCAAGCCTTCAGAAGACCAAGGGCCTTTCCTCCCATTGATGTCCTACAAGGCCAT  chr14

 71  CCTCTGCTACATATGTAGTTGGAGTCCATGGGTCCCTCAATGTGTACTCTTTGGTTGGCTGTTTAATCTC  160-9B_T7
     CCTCTGCTACATATGTAGTTGGAGTCCATGGGTCCCTCAATGTGTACTCTTTGGTTGGCTGTTTAATCTC  chr14

141  TGAGAGCTCTGGCGAATCTGGATGATTGATATTATTGTTCTTCCTATGGGGTTGCAAACCCCTTCAACTC  160-9B_T7
     TGAGAGCTCTGGCGAATCTGGATGATTGATATTATTGTTCTTCCTATGGGGTTGCAAACCCCTTCAACTC  chr14

211  CTTCAGTCCTTTCATTTACTCCATTGGGGACCCCAAGCTCAGTCCAATGGTTGGCTGTGAGCATCTGCCT  160-9B_T7
     CTTCAGTCCTTTCATTTACTCCATTGGGGACCCCAAGCTCAGTCCAATGGTTGGCTGTGAGCATCTGCCT  chr14

281  CTGTATATGTCAGGCTCTATCAGAGCTTCTCAGGAGACAGTTATACCAGGCTCCTGTCAGCATGCACTTC  160-9B_T7
     CTGTATATGTCAGGCTCTATCAGAGCTTCTCAGGAGACAGTTATATCAGGCTCCTGTCAGCATGCACTTT  chr14

351  TTGACATCAGCAATA  160-9B_T7
     TAAACATCAGCAATA  chr14
```

>182-12B_SP6 vs. chr17: 15,428,227-15,428,613 (-), 387 bp overlap

```
  1   CAAGCTAGACCCATCAGAGAGGAGAGTCTCTCAATTGAGAAAATGTTGTGTGTAGTTTTATGCCCTACCC   182-12B_SP6
      CAAGCTAGACCCATCAGAGAGGAGAGTCTCTCAATTGAGAAAATGTTGTGTGTAGTTTTATGCCCTACCC   chr17

 71   ACTGATATTCTGGGAGTTGACTTGGCTACCAGCCCCCACTGGGTATCCCTTGAATCCTCAGACAAAGGTC   182-12B_SP6
      ACTGATATTCTGGGAGTTGACTTGGCTACCAGCCCCCACTGGGTATCCCTTGAATCCTCAGACAAAGGTC   chr17

141   ACACACACACACACACACACACACACACACACACACACACACACACACAGAGTTTATTCCTTTTCAACTTGC   182-12B_SP6
      ACACACACACACACACACACACACACACACACACACACACACACACACAGAGTTTATTCCTTTTCAACTTGC   chr17

211   CTTAGGACTCAATTGTTGTTTGCCAAACTCCCTAACCACCCACCTGTAGTAGTGGCCACAGGCCCCAGTT   182-12B_SP6
      CTTAGGACTCAATTGTTGTTTGCCAAACTCCCTAACCACCCACCTGTAGTAGTGGCCACAGGCCCCAGTT   chr17

281   CCTCCCAAGACCTCACATGGCTGCTGGCTTTTTCTGCCTCCACAGCATGGCCAATGTACCTTATCTCTTC   182-12B_SP6
      CCTCCCAAGACCTCACATGGCTGCTGGCTTTTTCTGCCTCCACAGCATGGCCAATATACCTTATCTCTTC   chr17

351   CCTTGCATCTGCTTTTTCCTTCTGGGACCTGGAATTC   182-12B_SP6
      CCTTGCATCTGCTTTTTCCTTCTGGGACCTGGAATTC   chr17
```

>192-6C_T7 vs. chr17: 33,999,368-33,999,883 (-), 515 bp overlap

```
  1   CCTGTCTCTGTGTTGGGCTTTTGGGG-CTCTCCATGTCCTGTGTCCCCACTCCCTCTGACTTCTTTGTCC   192-6C_T7
      CCTGTCTCTGTATTGGGCTTTTGGGGGCTCTCCATGTCCTGTGTCCCCACTCCCTCTGACTTCTTTGTCC   chr17

 70   TGGGAAGCTGTCCTTTGCTGCTGGGGTCCCGAAGGTTTATGCCAGGTACAGGAGAGAAGATGCACTAGGT   192-6C_T7
      TGGGAAGCTGTCCTTTGCTGCTGGGGTCCCGAAGGTTTATGCCAGGTACAGGAGAGAAGATGCACTAGGT   chr17

140   GCCCAGCATTCAGTTTGACTCGATCTTAGCAGAAGTTAAGCCAGGGGCTGCTGAACACCACGTGAGTATA   192-6C_T7
      GCCCAGCATTCAGTCTGACTCGCTCTTGGCAGAAGTGAAGCCAGGGGCTGCCGGGCACCACGTGAGTATA   chr17

210   GTGTCCACTGTCTCTACTTGCAGTTCTGCTGAGTCCCAACCCAGGCTGCACCAGACCCAGCTTCACAGGA   192-6C_T7
      GTGTCCACTGTCTCTACTTGCAGTTCTGCTGAGTCCCAACCCAGGCTGCACCAGACCCAGCTTCACAGGA   chr17

280   CAGGCTGTGTGCACCACAGCTGCTACCATTGGTCCCCATTCACAAAGCGCTAATGACTTAAGGGCTCAAA   192-6C_T7
      CAGGCTGTGTGCACCACAGCTGCTACCATTGGTCCCCATTCACAAAGCGCTAATGACTTAAGGGCTCAAA   chr17

350   TGTGCTCCATTGTGTAGAAAGCTTGCCCTTCTGGAGAACAATACCCTTCCCTATTCTGCAGCCTTTATAC   192-6C_T7
      TGTGCTCCATTGTGTAGAAAGCTTGCCCTTCCGGAGAACAATGCCCTTCCCTATTCTGCAGCCTTTATAC   chr17

420   AGCCCTGTGAGCCTTCCAGGAGCCCAGGTAAGACAGGGTCCCCCAGAGGCTGGTTGCCTCTCAGAGGCAG   192-6C_T7
      AGCCCTGTGAGCCTTCCAGGAGCCCAGGTAAGACAGGGTCCCCCAGAGACTGGTTGCCTCTCAGAGGCAG   chr17

490   ATTGATGGTACATCTACAATGAATTC   192-6C_T7
      ATTGATGGTACATCTACAATGAATTC   chr17
```

>200-9C_T7 vs. chr6: 99,758,420-99,758,598 (+), 180 bp overlap

```
  1   GAATTCAAACCTCTTATCCATTCCTTGGTCCATATTCCCTTTCCTTAGTGGTTTTACTCTGAAATGTTGC   200-9C_T7
      GAATTCAAACCTCTTATCCATTCCTTGGTCCATATTCCCTTTCCTTAGTGGTTTTACTCTGAAATGTTGC   chr6

 71   ATGACCCAACAAAGTCCTGCCANNGCATAGTGGTGCAGATGCGAACTTCAGAACTCTATAGTGGCTGAAG   200-9C_T7
      ATGACCCAACAAAGTCCTGCCAG-GCATAGTGGTGCAGATGCGAACTTCAGAACTCTATAGTGGCTGAAG   chr6

141   AAGGAGGTAGATCCTAAGTTTGATGTCAGACTAAGTTACA   200-9C_T7
      AAGGAGGTAGATCCTAAGTTTGATGTCAGACGAAGTAACA   chr6
```

>245-11P_SP6 vs. chr17: 12,000,500-12,000,962 (+), 465 bp overlap

```
  1  GAATTCTATCCCAAACACATGCTTAAAAAGCAGTTGTGCGGAGAAGTGGAGAAGTGAATCAGCAGTTAAG  245-11P_SP6
     GAATTCTATCCCAAACACATGCTTAAAAAGCAGTGGTGCGGAGAAGTGGAGAAGTGAATCAGCAGTTAAG  chr17

 71  AGCAATTTTGGCTTTGGAAGACGACCTGAGTTCAATATCCAGCACCAACATGGTGGCTAACCACCAGCCA  245-11P_SP6
     AGCAATTTTGGCTTTGGAAGACGACCTGAGTTCAATATCCAGCACCAACATGGTGGCTAACCACCAGCCA  chr17

141  TAACTCCAGTTCTAGGGGATCCTATGCCTTCTTTGGGCTTCTGCGGGCATCCGGCATATATATGGTTCAC  245-11P_SP6
     TAACTCCAGTTCTAGGGGATCCTATGCCTTCTTTGGGCTTCTGCGGGCATCCGGCATATATATGGTTCAC  chr17

211  AGACACACATGCAGGAAAAACACTCATACACATAAAATATGTAAATCATAAAATACATAAATAAAAAAAA  245-11P_SP6
     AGACACACATGCAGGAAAAACACTCATACACATAAAATATGTAAATCATAAAATACATAAATAAAAAAAA  chr17

281  TTAAACCAAGCATGGTTATGGATTCTTGGATTTCAACACTAAGGAGGCAGAACCCCCTGGGATTCAGTAT  245-11P_SP6
     TTAAACCAAGCATGATTATGGATTCTTGGATTTCAACACTAAGGAGGCAGAACCCC-TGGGATTCAGTAT  chr17

351  CAGGGCAGCCAGCCTGGCCTACTTAATGAGTCTCAGGCCAGGTAAGAGACCATATCTCAAGAAGGAAGAA  245-11P_SP6
     CAGGGCAGCCAGCCTGGCCTACTTAATGAGTCTCAGGCCAGGTA-GAGACCATATCTCAAGAAGGAAGAA  chr17

421  CATCATCTGAGGAAAGGTGCCTGAGGTTGGCCTCTGTTTTCCATA  245-11P_SP6
     CATCATCTGAGGAAAGGTGCCTGAGGTTGGCCTCTGTTTTCCATA  chr17
```

>245-11P_T7 vs. chr17: 12,186,842-12,187,321 (-), 480 bp overlap

```
  1  TATGTTAGCTATTTTGCTAGATTGCTGGTTGGAGATATCCTGATGATGGAGATTGGTCTTACCCCAAGAA  245-11P_T7
     TATGTTAGCTATTTTGCTAGATTGCTGGTTAGAGATATCCTGATGATGGAGATTGGTCTTGCCCCAAGAA  chr17

 71  ACCCAAGGCTGCTAGTCAGCAGGAAGTAGTCTAAGGAGGCCTACACCAGGAGAGTCTACACCTTCTCTGG  245-11P_T7
     ACCCAAGGCTGCTAGTCAGCAAGAAGTAGTCTAAGGAGGCCTACACCAGGAGAATCTACACCTTCTTTGG  chr17

141  TCTTCTTTCTCCCCTACCTAGTGTTGGGTAGTTGGACGGGATTGGGGTAGAATAAGGGTTGGAAGGATGG  245-11P_T7
     TCTTCTTTCTCCCCTACCTAGTGTTGGGTAGTTGGACGGGATTGGGGTAGAATAAGGGTTGGAAGGATGG  chr17

211  TAGATACTAGAACCCAATAAAGTAGCCCAAGAAGCAAGTCATCAAGAATTAATATGACGACATGTACACT  245-11P_T7
     TAGATACTAGAACCCAATAAAGTAGCCCAAGAAGCAAGTCAACAAGAATTAATATGACGACGTGTACACT  chr17

281  GGATAGTTTTCTGACAACTTGACACAAGCTAGAATATCTGAAAGAAGGAACCTCAACTGAAGGAAAAAAC  245-11P_T7
     GGATAGTTTTCTGACAACTTGACACAAGCTAGAATATCTGAAAGAAGGAACCTCAACTGAAGGAAAAAAC  chr17

351  TCAATAAAATCCATCTATAAGGCATTGTCTTAATGAGTGATTGATGGGTGAGGGCCCAGGTCATTTGGGG  245-11P_T7
     TCAATAAAATCCATATATAAGGCATTGTCTTAATGAGTGATTGATGGGTGAGGGCCCAGCTCATTTGGGA  chr17

421  TGGTGCTTGTAGTCCTAGGTTCTATAAGAAAGCAGGCTGAACAATTCATGTGGAGCAAGC  245-11P_T7
     TGGTGCTTGTAGTCCTGGGTTCTATAAGAAAGCAGGCTGAACAATTCATGTAGAGCAAGC  chr17
```

>245-9D_SP6 vs. chr4: 92,886,305-92,886,528 (+), 223 bp overlap

```
  1  AGGAACTACGAACTTCTTGCGACTGTGACTGTGGGTGACATTCCTGAACTTGGGTNNCAGGTGTTTGTTG  245-9D_SP6
     AGGAACTACGAAATTCTTGCGACTGTGACTGTGGGTGAAATTCCTGAACTTGGGTCTCAGGTGTTTGTTG  chr4

 71  NTGTGGGTTTTTTTTTAATGCGTGTGTAATGTGAAAATAATCAGAATCTATTAAATAGNGGT---CAGTG  245-9D_SP6
     TTGTGGGTTTTTTTTTAATGTGTGTGTAATGTGAAAATAATCAGAATCTATTAAATAGTGGTGGTCAGTG  chr4

138  CTTACACCGGCATTTGTAACTACTTCCCGACCCACAGATCCTCCTACGGAGATCGGGGAAACCACCAAGT  245-9D_SP6
     CTTACACCGGCATTTGTAACTACTTCCCGACCCACAGATCCTC-TACGGAGATCGGGGAAACCAC-AAGT  chr4

208  AAAAGAAACCGCGAAA  245-9D_SP6
     AAAAGAAACCGCGAAA  chr4
```

>245-9D_T7 vs. chr4: 93,023,688-93,024,308 (-), 621 bp overlap

```
  1  AAACAGTCATATACTTAAGAATAAACAAATTGATTAAAATAAAAATTACTTATGTGCCCCACCCGGCAAA  245-9D_T7
     AAACAGTCATATACATAAGAATAAACAAATTGATTAAAATAAAAATTACTTATGTGCCCCACCCGGCAAA  chr4
```

```
 71   AAAGAAAACTTGCTAACACTCTTGAGACCAGGAGCTGACCCCACACTGCCTCTCCGATGGATTAACATTC   245-9D_T7
      AAAGAAAACTTGCTAACACTCTTGAGACCAGGAGCTGACCCCACACTGCCTCTCCGATGGATTAACATTC   chr4

141   CTCTGCTAATTGCCCCTTGAAAATCTTTTTACCTTTATCTTTACCTAAAATTCTAATGAAAAACTGCACA   245-9D_T7
      CTCTGCTAATTGCCCCTTGAAAATCTTTTTACCTTTATCTTTACCTAAAATTCTAATGAAAAACTGCACA   chr4

211   TAAACGATTAAGATATTAACTGCTAGAGTTGGCCACCTAGTTAACAGAGTGCTCGGTAGCATTAGGAACA   245-9D_T7
      TAAACGATTAAGATATTAACTGCTAGAGTTGGCCACCTAGTTAACAGAGTGCTCAGTAGCATTAGGAACA   chr4

281   GTTACAGGAGCTTGCCTGGTTCTGAGAACTGGCTTTCTGACACCTGTGTTTCTTGGGCTGTCTTCATGAG   245-9D_T7
      GTTACAGGAGCTTGCCTGGTTCTGAGAACTGGCTTTCTGACACCTGTGTTTCTTGGGCTGTCTTCATGAG   chr4

351   CACTGGAGTTGGAATTGTGTTACCTGTACCCTATCAGAGTCTATTTGGAATAATAGTATATGATATTGTT   245-9D_T7
      CACTGGAGTTGGAATTGTGTCACCTGTACCCTATCAGAGTCTATTTGGAATAATAGTATATGATATTGTT   chr4

421   GGGATCAGGAAACGAGTGTCTTGAAATGAGTCCCGGAGACAACACTGGTTGAAATTTAATTCACACCATC   245-9D_T7
      GGGATCAGGAAAGGAGTGTCTTGAAATGAGTCCCGGAGACAACACTGGTTGAAATTTAATTCACACCATC   chr4

491   CAAGGCAGGCTGCATGTAGAATTCCTAGAAACAGCTTTGGTAGAAGAGAGCAGAAAGGAGACATTCTAAA   245-9D_T7
      CAAGGCAGGCTGCATGTAGAATTCCTAGAAACAGCTTTGGTAGAAGAGAGCAGAAAGGAGACATTCTAAA   chr4

561   GAGATACTTAGCCAGGGTAGGATTTGGAGAAGGATTGTGAGTGGGGATGACAGGGAGGGCC   245-9D_T7
      GAGATACTTAGCCAGGGTAGGATTTGGAGAAGGATTGTGAGTGGGGAAGACAGGGAGGGCC   chr4
```

```
>255-3L_SP6 vs. chrX: 94,890,859-94,891,345 (-), 475 bp overlap
```

```
  1   AAAGGGCCGAGATGGGGCCCGCAAGCTAACACCTCAGGGTCAGGAAACACATCTGGACAGGATCGCTGAA   255-3L_SP6
      AAAGGGCCGAGATGGGGGCCGCAAGCTAACACCTCAGGGACAGGAAACACATCTGGACAGGATCGCTGAA   chrX

 71   CAGGTGGCAGCTGCCAACAAGAAGCATTAGAACAAATAATACTGGGTTAATAAATTGCCTCATTCATAAA   255-3L_SP6
      CAGGTGGCAGCTGCCAACAAGAAGCATTAGAAAAAATAATACTGGGTTAATAAATTGCCTCATTCATAAA   chrX

141   AAAGAAAAGAATAAAGAAAGAGAAGCCTATAAACATACAAGAAGCTTATAGAGTACCAATTATAACCAAA   255-3L_SP6
      AAAGAAAAGAATAAAGAAAGAGAAGCCTATAAACATACAAGAAGCTTATAGAGTACCAATTATACCCAAA   chrX

211   AAAAAAAAAAAAAAAAA-------------CAAAACAAAAAACAAAACCCTCCTGGCATGTAATAATCAA   255-3L_SP6
      AAAAAAAAAAAAAAAAAAAAAAAAACCAAACAAAACAAAAAACAAAACCCTCCTGGCATGTAATAATCAA   chrX

268   ATGTAAATACAAATAATAATGTTTTTGGGGAAGATGTAATGATCCATTCCATTAATACGAGCACTTGGGA   255-3L_SP6
      ATGTAAATACAAATAATAATGTTTT-GGAGAAGATGTAATGATCCATTCCATTAATACGAGCACTTGGGA   chrX

338   AGCAGAGCCATGGTGGATGGATCTCAGAGGAGTGTGAACTTAATGTGGGCCTCTGCTGTAGTAGCAAGTG   255-3L_SP6
      AGCAGAGCCATGGTGGATGGGTCTCAGAGGAGTGTGAACTTAATGTGGGCCTCTGCTGTAGCAGCAAGTG   chrX

408   TTCTTAGCTGCTAAGCCATCTCTGTGGCTTCTTTTATTCCAAGGACTGTTTAAGCTGACCTGGAATTC   255-3L_SP6
      TTCTTAGCTGCTAAGCCATCTCTGTGGCTTCTTTTATTCCAAGGACTGTTTAAGCTGACCTGGAATTC   chrX
```

```
>255-3L_T7 vs. chrX: 94,756,622-94,757,143 (+), 524 bp overlap
```

```
  1   GTTTTACAGAGACATATGAATGTCCACAACAGTATTTACATAATATATAAATGATGAAAGGAATCCAAAT   255-3L_T7
      GTTTTACAGAGACATATGAATGTCCACAACAGTATTTACATAATATATAAATGATGAAAGGAATCCAAAT   chrX

 71   GTCATTACCAGGTGAGTGGATAAGAAAAATATGATCTACTGCAACAATAAAATACTATTTATCCATTAAA   255-3L_T7
      GTCATTACCAGGTGAGTGGATAAGAAAAATATGATCTACTGCAACAATAAAATACTATTTATCCATTAAA   chrX

141   AGGAATGAAGCTCTAATAAATGACACAATATGAAGAACCTAGAGGACACACTAAGCAAAAGGTGTCAGAA   255-3L_T7
      AGGAATGAAGCTCTAATAAATGACACAATATGAAGAACCTAGAGGACACACTAAGCAAAAGGTGTCAGAA   chrX

211   GCAATGTATTTTATAATATAGTTTATTTGAAAAGCCCTGCTAAAGAAATACTAACTCATAACACAGACTA   255-3L_T7
      GCAATGTATTTTATAATATAGTTTATTTGAAAAGCCCTGCTAAAGAAATACTAACTCATAACACAGACTA   chrX

281   ATTGTTGCNNTAGGATCGGGGATGAGAATTGGGGCAGGGGCTATTGAAAAACGAGGCAATGGAAATATTC   255-3L_T7
      ATTGTTGCA-TAGGATCGGGGATGAGAATTGGGGCAGGGGCTATTGAAAAACGAGGCAATGGAAATATTC   chrX
```

```
351  TAAAATTAGTTTCTGATGATGACTGCACCATTCTGTAAATTGACTAATATCACTAACATGCATACTTTAA  255-3L_T7
     TAAAATTAGTTTCTGATGATGACTGCACCATTCTGTAAATTGACTAATATCACTAACATGCATACTTTAA  chrX

421  CAAACAACACTATCTTTTAAAACACTCTCAGACCATTAAACCTGAGACTTTGTGCCTCAGTCAGCCATCT  255-3L_T7
     CAAACAACACTATCTTTTAAAACCCTCTCAGACCATTAAACCTGAGACTTTGTGCCTCAGTCAGCCATCT  chrX

491  ATTAGCCCGAGAGAATGACCCTCATGACCTTCTT  255-3L_T7
     ATTAGCC-GAGAGAATGACCCTCATGACCTTCTT  chrX
```

>257-22B_SP6 vs. chrX: 130,761,904-130,762,049 (+), 174 bp overlap

```
  1  TCCCCCTGNCTCTGTCATCACCGAGNGCCTGGGATTACAAGGGGTGCGCCACCACGNCNGGCTCAATTTA  257-22B_SP6
     TCCACCTGCCTCTG-CCTC-CCGAGTGC-TGGGATTA-AAGGCGTGCGCCACCACGCCCGGCTCAATTTA  chrX

 71  TCTTTTG-GNAANGCANTTGNGTCCATCATANAGAGTCTTCCTGTCTCTTACATTANCCANATTTTGAAA  257-22B_SP6
     TCTTTTCAGTAATACATTTGTCTCCATCACACACATTCATCCTGTCTCTTACATT-TCCATATTTTGACA  chrX

140  TTTTTCGTTGACTATGAAGTGTATTTNTTTT-ACAT  257-22B_SP6
     TTTTTTGTTGACTATGAAGTGTATTTTTTTTTACAT  chrX
```

>257-22B_T7 vs. chrX: 130,885,586-130,886,114 (-), 539 bp overlap

```
  1  TTGGAATTCACTGTAATAAGGCTGTACTTGAATGTCCCTCACATCACCAAAGGATTTTTACCTCCATAGT  257-22B_T7
     TTGGAATTCACTGTAATAAGGCT----------GTCCCTCACATCACCAAAGGATTTTTACCTCCATAGT  chrX

 71  AGATAAGTTAAGAGTTGACAGTTCTGCTGCACCAAGGAAAGAATTGTAGGCACCATTTTTGGATACAGAT  257-22B_T7
     AGATAAGTTAAGAGTTGACAGTTCTGCTGCACCAAGGAAAGAATTGTAGGCACCATTTTTGGATACAGAT  chrX

141  TTCCTGCTACGGTCAAAGCTTTTTGACTGGAGTGATTGTCGTCATTCTCAGCCCGCAGGGAACAGGTTAC  257-22B_T7
     TTCCTGCTACGGTCAAAGCTTTTTGACTGGAGTGATTGTCGTCATTCTCAGCCCACAGGGAACAGGTTAC  chrX

211  ATTCATTTAAGAAATGGTGTGTGTATTAAGATGGTCTATGTATGAAGTCATTCACCAACTGTTTCAATGT  257-22B_T7
     ATTCATTTAAGAAATGATGTGTGTATTAAGATGGTCTACCCATGAAGTCATTCACCAACTGTTTCAATGA  chrX

281  ACAATGTTCTGCTTGGAGGGTGGCACGAACATTAGGTGAGGGTAAGATTCTGGTTCATTGGCTGTGATGA  257-22B_T7
     ACAATGTTCTGCTTGGAGGGTGGCACGAACATTAGGTGAGGGTAAGATTCTGGTTCATTGGCTGTGATGA  chrX

351  TTTTGAAAAGCATTCATCTCAGAATAAGAGTAACTTATAAAGTCCTCTTCTTCAATGTTTTCTATGGTAT  257-22B_T7
     TTTTGAAAAGCATTCATCTCAGAATAAGAGTAACTTATAAAGTCCTCTTCTTCAATGTTTTCTATGGTAT  chrX

421  CTTCTGCACTTGAGATTCTCTCTTCTATCTCTTGTATTCTGTTGGTGATGCTTGCATCTATGACTCCTAG  257-22B_T7
     CTTCTGCACTTGAGATTCTCTCTTCTATCTCTTGTATTCTGTTGGTGATGCTTGCATCTATGACTCCTAG  chrX

491  GTTTTCTAACTCCAGGGTTGTCTCCTTTTGTGATTTCTTTATTGTTTCT  257-22B_T7
     GTTTTCTAACTCCAGGGTTGTCTCCTTTTGTGATTTCTTTATTGTTTCT  chrX
```

>262-11G_T7 vs. chr12: 35,192,049-35,192,443 (-), 397 bp overlap

```
  1  CTCAGCTCGACTGGAGGCCAGGCTATATGTGCATAGCCCATTGCCCCACATTCCTCCACCTAAGAAGATG  262-11G_T7
     CTCAGCTCGACTGGAGGCCAGGCTATATGTGCATAGCCCATTGCCCCACATTCCTCCACCTAAGAAGATG  chr12

 71  CCAAATACTTAGAGAGACGACTTACAGGTATCCTGTGATGCAGCTGCTCTCTCTACCCTTATTTACCTTC  262-11G_T7
     CCAAATACTTACAGAGACGACTTACAGGTATCCTGTGATGCAGCTGCTCTCTCTACCCTTATTTACCTTC  chr12

141  ATCCTCTTATTTATATGTATGGCCAAAAAGGCAAATGTCTACAGAAAAAAAAACAAAACAAAAACCCTAG  262-11G_T7
     ATCCTCTTATTTATATGTATGGCCAAAAAGGCAAATGTCTACAGAAAAAAAAA-AAAACAAAA-CCCTAG  chr12

211  GCACCTCTTTAAAGAATTGTTTTAGAAAAAGTAACAAGAGCTGAGAAGCTGTCTCAGTCAGAAAAGCACT  262-11G_T7
     GCACCTCTTTAAAGAATTGTTTTAGAAAAAGTAACAAGAGCTGAGAAGCTGTCTCAGTCAGAAAAGCACT  chr12
```

```
281  TGTCACAAACATCAGGGCCTGAGTTCAAAACCAAGAACCTATGCAAACACCCTGGGCTTGTAGCATACCT  262-11G_T7
     TGTCACAAACATCAGGGCCTGAGTTCAAAACCAAGAACCTATGCAAACACCCTGGGCTTGTAGCATACCT  chr12

351  GTAATCCTAGCATTGTGGAAGTGAAGACAGGAAGACCGCTAGAATTC  262-11G_T7
     GTAATCCTAGCATTGTAGAAGTGAAGACAGGAAGACCCCTAGAATTC  chr12
```

>266-2E_SP6 vs. chr12: 35,090,782-35,091,223 (+), 442 bp overlap

```
  1  GAATTCAACTGATGACACCCCTAAAGAAATGTCAACTATGGGCGAGGTCAGGAATTCAACAGTGGAGCCT  266-2E_SP6
     GAATTCAACTGATGACACCCCTAAAGAAATGTCAACTATGGGCGAGGTCAGGAATTCAACAGTGGAGCCT  chr12

 71  TGTGATTGACTCTTCCATATGAACTCAAATGAGTGTCCACAGGTAAGGACTCCACTGTTGATTTAATGGA  266-2E_SP6
     TGTGATTGACTCTTCCATATGAACTCAAATGAGTGTCCACAGGTAAAGACTCCACTGTTGATTTAATGGA  chr12

141  TTAAAAAAATTAGTTACAATTAAACACATGAGAATCTGTGCCTAGAATGTTATAAGATCAATATGGAGAC  266-2E_SP6
     TTAAAAAAATTAGTTACAATTAAACACATGAGAATCTGTGCCTAGAATGTTATAAGATCAATATGGAGAC  chr12

211  CAGATTTAAATGATAAGACCACATGAGATCACCTTGGCCTGGAATACTCCAGTGCTTAGAGGCCAAGCAG  266-2E_SP6
     CAGATTTAAATGATAAGACCACATGAGATCACCTTGGCCTGGAATACTCCAGTGCTTAGAGGCCAAGCAG  chr12

281  AGTAAAGCCCAAACCTCGCTGGGAATGTGGAGACATCTAGCTCATCAGAAAAATATGCTCAACAAACAGT  266-2E_SP6
     AGTAAAGCCCTAACCTCTCTGGGAATGTGGAGACATCTAGCTCATCAGAAAAATATGCTCAACAAACAGT  chr12

351  ACACGTTGACTGTGGTAAATGCCTGCCTGTGGCTGGTTAAGAAAAACAAGATCAGAGAAGTGATCTTTGA  266-2E_SP6
     ACAAGTTGACTGTGGTAAATGCCTGCCTGTGGCTGGTTAAGAAAAACAAGATCAGAGAAGTGATCTTTGA  chr12

421  AAGCACTGTGCTCATTCACCCC  266-2E_SP6
     AAGCACTGTGCTCATTCACCCC  chr12
```

>266-2E_T7 vs. chr12: 35,199,619-35,200,180 (-), 561 bp overlap

```
  1  AAATGCGTGTATATCTAAATGTGATGAAGATATTTATGTGTTGAATAAAGAAAACTAAAATTAAATAGGA  266-2E_T7
     AAATGCGTGTATATCTAAATGTGATGAAGATATTTATGTGTTGAATAAAGAAAACTAAAATTAAGTAGGA  chr12

 71  CGTGTTGCTTACAATTTGGCAGTAGAGGCTCCGGGTGACAAATTAAAAGCTTATATTAAAAATTAACATA  266-2E_T7
     CGTGTTGCATACAATTTGGCAGTAGAGGCTCCGGGTGACAAATTAAAAGCTTATATAAAAAATTAACATA  chr12

141  AATGTAACCTGACGATGTGGAGCATGGCCTGGATTTGTATCCAGGAGCAACAAAGTGTCCTTAAGACACT  266-2E_T7
     AATGTAACCTGACGATGTGGAACATGCCCTGGATTTGTATCCAGGAGCAACAAAGTGTCCTTAAGACACT  chr12

211  GGGTCTCCCACTGGCTTCTGGAAATTCTGATAGCTAGGATGCGCTAACTCCAGAAGCAACAGTTATTAAA  266-2E_T7
     GGGTCTCCCACTGGCTTCTGGAAATTCTGATAGCTAGGATGCGCTAACTCCAGAAGCAACAGTTATTAAA  chr12

281  GACGCACACACAAAGTAGAGACAGACCGGCCTGAAGTACTTACTTATTAGAAAGATGGCGAAGAAGACCA  266-2E_T7
     GACGCACACACAAAGTAGAGACAGACCTGCCTGAAGTACTTACTTATTAGAAAGATGGCGAAGAAGACCA  chr12

351  GAGCCAGGCTGACCGTCAACACCAGGAGCAGCCCCACAAAAAACAGCCCCCGCCTTCCATTTCCTGGGGC  266-2E_T7
     GAGCCAGGCTGACCGTCAACACCAGGAGCAGCCCCACAAAAAACAGCCCCCGCCTTCCATTTCCTGGGGC  chr12

421  TGTGCCAGCATTGGGGATTTTGTCCTCTAAAGCTGATGGGAAACAAAGTGACACAATCTCCGCATCTCAC  266-2E_T7
     TGTGCCAGCATTGGGGATTTTGTCCTCTAAAGCTGATGGGAAACAAAGTGACAGAATCTCCGCATCTCAC  chr12

491  AGAGATGTGATGCACACACTCATTTTCCCTAGACAGCCCTGAAAAGTGACTTCTGCATTCGGAA-TGCCT  266-2E_T7
     AGAGATGTGATGCACACACTCATTTTCCCTAGACAGCCCTAAAAAGTGACTTCTGCATTCGGAAATGCCT  chr12

560  TT  266-2E_T7
     TT  chr12
```

>269-9H_SP6 vs. chrX: 94,817,775-94,818,021 (+), 247 bp overlap

```
  1  AAAAAGAAGTACACAGGACCTACTCCTTCATGCCAATACTCACTAGATCACAGAGATTTCCTTAATACTT  269-9H_SP6
     AAAAAGAAGTACACAGGACCTACTCCTTCATGCCAATACTCACTAGATCACAGAGATTTCCTTAATACTT  chrX
```

```
 71  CTACCCTAGCTGAAGGAAAATGTAGAGGTCAATGTTTTCTAAGACCTCCATGGAAGATAGGCATTTAGGA  269-9H_SP6
     TTACCCTAGCTGAAGGAAAATGTAGAGGTCAATGTTTTCTAAGACCTCCATGGAAGATAGGCATTTAGGA  chrX

141  TACTACAGAGGTGTGTCATTCTCTGGCTCTACCCATTCCCAATACCGGAGGCTCTCCAGACTCCGCCCCC  269-9H_SP6
     TACTACAGAGGTGTGTCATTCTCTGGTTCTACCCATTCCCAATACCCGAGGCTCTCCAGACTCCGCCCCC  chrX

211  TCAACTTGACCCCACCCCAGCACGAGCTGTGATAAAA  269-9H_SP6
     TCAACTTGACCCCACCCCAGCACGAGCTGTGATAAAA  chrX
```

```
>269-9H_T7 vs. chrX: 94,967,482-94,968,088 (-), 613 bp overlap
```

```
  1  GGCAACTGTCTTAGCGGCATGGTTCAGGCAGCAGGAGGCCACACATGATTCTGACACTGTGCCATCTGGA  269-9H_T7
     GGCAACTGTCTTAGCGGCATGGTTCAGGCAGCAGGAGGCCACACACGATTCTGACACTGTGCCATCTGGA  chrX

 71  GGAAGAGAATGAGAGAAACTTGCGTCATCATCTTTTCATCGCAGTGCAGCGACCTGGCACAAAACACCTA  269-9H_T7
     GGAAGAGAATGAGAGAAACTTGCGTCATCATCTTTTCATCGCAGTGCAGCGACCTGGCACAAAACACCTA  chrX

141  GAAAGGGAATGTGCAATTGGCAAATGACACGATTACATTTTCCCACAGTGGAAAGGCGTGAAGGACATGG  269-9H_T7
     GAAAGGGAATGTGCGATTGGCAAATGACAAGATTACATTTTCCCACAGTGGAAAGGCGTGAAGGACATGG  chrX

211  ACACACACACACACACACACACACACACAACACACAGGCAAAGCTTGACACATTTCAGAAAATCTTTAAA  269-9H_T7
     ACACACACACACACACACACAC------AACACACAGGCAAAGCTTGACACATTTCAGAAAATCTTTAAA  chrX

281  ATTTTCTGTTTTCTGTGTTTGCATATGTTAGTGTAATATGCATGTGCACCACATTTGTATAGGTGAATGT  269-9H_T7
     ATTTTCTGTTTTCTGTGTTTGCATATGTTAGTGTAATATGCATGTGCACCACATTTGTATAGGTGAATGT  chrX

351  AAAAGTCAAAAGCTGCATCAAGTTACCTGACTGTGGAGTTACAGATGGTTGGGAGCCATCTTGTGGGTCC  269-9H_T7
     AAAAGTCAAAAGCTGCATCAAGTTACTTGACTGTGGAGTTACAGATGGTTGGGAGCCATCTTGTGGGTCC  chrX

421  ATTACAGCTCCTATTCCTAGAAATCGTTTTTTAAGGCTTTTAATTCTTTTTTCCTGCGCCAAAAAAATATC  269-9H_T7
     ATTACAGCTCCTATTCCTAGAAATCGTTTTTTAAGGCTTTTAATTCTTTTTTCCTGCGCCAAAAAAATATC  chrX

491  TGTAAAGATTGCAAGAGGAGGCTCCTTCTCCAAATGGACAGTTGCCCACGCAGGGCTACAAGAACAAAGT  269-9H_T7
     TGTAAAGATTGCAAGAGGAGGCTCCTTCTCCAAATGGACAGTTGCCCATGCAGGGCTACAAGAACAAAGT  chrX

561  CAGGAAAAACATGAAACTATCAGTCACAAAATAAACTCCCAGAACCCTTAGAA  269-9H_T7
     CAGGAAAAACATGAAACTATCAGTCACAAAATAAACTCCCAGAACCCTTAGAA  chrX
```

```
>271-12L_SP6 vs. chrX: 94,854,496-94,854,839 (+), 344 bp overlap
```

```
  1  GAATTCTCTAAAACTTCCAGAAGGAATAATTTCAATTTAATTTCAAATAATAATTTCAATTTCAACTAGG  271-12L_SP6
     GAATTCTCTAAAACTTACAGAAGGAATAATTTCAATTTAATTTCAAATAATAATTTCAATTTCAACTAGG  chrX

 71  TGTCTCACACTGAACTTCTGACTTCTAAAATTATAAGGTTATAGATCTGTGTTGCTTTAAGCCACCAAGT  271-12L_SP6
     TGTCTCACACTGAACTTCTGACTTCTAAAATTATAAGGTTATAGATCTGTGTTGCTTTAAGCCACCAAGT  chrX

141  TTGTTGGGGGCTTAAACAAATACAGCACTGTAGTGCTACCTGTGTCTCTGTATCTTCGTAGAGGTTAAGG  271-12L_SP6
     TTGTTGGGGGCTTAAACAAATACAGCACTGTAGTGCTACCTGTGTCTCTGTATCTTCGTAGAGGTTAAGG  chrX

211  TGGTTATGAAGACATCCAGAAAAGGAATCATGAGATAGGCTAAGGCACAGAGTATGTCTTTTATTTACAG  271-12L_SP6
     TGGTTATGAAGACATCCAGAAAAGGAATCATGAGATAGGCTAAGGCACAGAGTATGTCTTTTATTTACAG  chrX

281  TTTCTTTGTAGAACACCTTTACCTAATGTCTTAATCAGCTAATTAGCATAGTAAAATACAACTT  271-12L_SP6
     TTTCTTTGTAGAACACCTTTACCTAATGTCTTAATCAGCTAATTAGCATAGTAAAATACAACTT  chrX
```

```
>271-12L_T7 vs. chrX: 94,985,811-94,986,461 (-), 616 bp overlap
```

```
  1  TAACCATTCCAAAGACCTTCCTATTATTTCCAGCTTTACTATGACTAACTAAATGACCTGGACTCATTTC  271-12L_T7
     TAACCATTCCAAAGACCTTCCTATTATTTCCAGCTTTACTATGACTAAGTAAATGACCTGGACTCATTTC  chrX
```

```
 71  CACATAGTAGTTCACCGCTTCTCATCTGTACAGGTACCTGAACATGTTTAAATAACTAAATTTAGGGATA  271-12L_T7
     CACATAGTAGTTCACCGCTTCTCATCTGTACAGGTACCTGAACATGTTTAAATAACTAAATTTAGGGATA  chrX

141  AAATTAGACTTCTTTATATGCCTTTAATAACTATCCTAAAGTTGGGCTGACATACTGGCATACACCTTTA  271-12L_T7
     AAATTAGACTTCTTTATATGCCTTTAATAACTATCTTAAAGTTGGGCTGACATACTGGCATACACCTTTA  chrX

211  GTCTGAGCACTTCAGAGGCAGAAGCAGGTGTATCTCTATGAGTTGGAGGCCAGCTTGGCTACACAGTGAG  271-12L_T7
     GTCTGAGCACTTCAGAGGCAGAAGCAGGCGTATCTCTATGAGTTGGAGGCCAGCTTGACTACACAGTGAG  chrX

281  TTCTAGGGCACCAGAACTACATAATGAGACCCTTTCCCCCACCCTAATGTTCTAAAACTTCTGTCAAATT  271-12L_T7
     TTCTAGGGCACCAGAACTACATAATGAGACCCTTTCCCCCACCCTAATGTTCTAAAACTTCTGTCAAATT  chrX

351  TACAGAAATCAGCATATATACTTTATAACTTTATAATTTCTGCGTGCTAAATTGAAATGATTTTATATAA  271-12L_T7
     TACAGAAATCAGCATATATACTTTATAACTTTATAATTTCTGCGTGCTAAATTGAAATGATTTTATATAA  chrX

421  TCCTCAATAGCAAATTTTTTCAAAAAGCAAATACAGAGGCTCAGCTGAAATTTTACAGAAGAGGAAGAG-  271-12L_T7
     TCCTCAATAGCAAATTTTTTCAAAAAGCAAATACAGAGGCTCAGCTGGAATTTTACAGAAGAGGAAAAGC  chrX

490  ---------------------------------AAAAAGGAAAAGCATATTTGTGAAGTGGAGGACCAAC  271-12L_T7
     ATATTTGTGCATAAAATAAAAGCATATTTACAGAAAAAGGAAAAGCATATTTGTGAAGTGGAGGACCAAC  chrX

527  TGCATACGTAGCCCAATCCCGTATCTTCATATTTAAGTGAATTTGAAGGATTTTATTGTAAGATGTAAAG  271-12L_T7
     TGCATACGTAGCCCAATCCCGTATCTTCATATTTAAGTGAATTTGAAGGATTTTATTGTAAGATGTAAAG  chrX

597  AATCCTGTCCTG-TAGAATTC  271-12L_T7
     AATCCTGTCCTGATAGAATTC  chrX
```

>272-12L_SP6 vs. chr3: 147,530,230-147,530,623 (+), 394 bp overlap

```
  1  GAATTCTTCTTTGTAGAATGTACAGAGATGAGATATAAATGTGACCTCTTGACTTACCATGTTGGCCTGT  272-12L_SP6
     GAATTCTTCTTTGTAGAATGTACAGAGATGAGATATAAATGTGACCTCTTGACTTACCATGTTGGCCTGT  chr3

 71  GCCCAACTTTCTGTTATTTCCCTAGAAAGCTTGAAAATATATCAAAGACAAGAAGCAATGAAGAAATGTA  272-12L_SP6
     GCCCAACTTTCTGTTATTTCCCTAGAAAGCTTGAAAATATATCAAAGACAAGAAGCAATGAAGAAATGTA  chr3

141  CGTCAGCCATGGAATGCAGAGTTAGATCTTATAAAATGTCACCCATTTAGAAAACCAAAGATTTAACTAA  272-12L_SP6
     CGTCAGCCATGGAATGCAGAGTTAGATCTTATAAAATGTCACCCGTTTAGAAAACCAAAGATTTAACTAA  chr3

211  CGTTATTAGATGTGGATTAATCGGATGCATCATTCTTTCAGCTCTGCTCCTGTGGGATGCAGTTGCTAAG  272-12L_SP6
     CGTTATTAGATGTGGATTAATCGGATGCATCATTCTTTCAGCTCTGCTCCTGTGGGATGCAGTTGCTAAG  chr3

281  TGAGGATAATTAGAAGTGAAGGAGGCTTCTATGGGGAGCACCCAAGGGGAGAGATCTGAAGGAGGCAGGA  272-12L_SP6
     TGAGGATAATTAGAAGTGAAGGAGGCTTCTATGGGGAGCACCCAAGGGGAGAGATCTGAAGGAGGCAGGA  chr3

351  AGAGCATTTCAGCTACAGCACAGGTCAGACTTGTGAGCTGAGGG  272-12L_SP6
     AGAGCATTTCAGCTACAGCACAGGTCAGACTTGTGAGCTGAGGG  chr3
```

>272-12L_T7 vs. chr3: 147,663,827-147,664,390 (-), 565 bp overlap

```
  1  ACTGGTCTAGCCAAGCAGGATCAACTGGCTGGAGAGGCATGCCAGTGCCAAGACAAGGTCACTTTATAGT  272-12L_T7
     ACTGGTCTAGCCAAGCGGGATCAACTGGCTGGAGAGGCATGACAGCGCCAAGACAAGGTCACTTTATAGT  chr3

 71  AGAGGCCAAGATGCTGGTATTGCTTCTTTTGTTAGATACCTCTTTATAAAATAAGGCTCCAGTCAGGATG  272-12L_T7
     AGAGGCCAAGATGCTGGTATTGCTTCTTTTGTTAGATACCTCTTTATAAAATAAGGCTCCAGTCAGGATG  chr3

141  CTGTTACCTCCACACTGGGCAGTGAATGACATCCAGGGAAACCTTTGAAAGGGTCTTACTCTCCAATGTG  272-12L_T7
     CTGTTACCCCCACACTGGGCAGTGAATGACATCCAGGGAAACCTTTGAAAGGG--TTACTCTCCAATGTG  chr3

211  TGGACACTGAGGAAAATGGCAGGAACTCCGATTGATGAAAACCTGTTAGCACATCCTGCTAGAAAATCAT  272-12L_T7
     TGGACATTGAGGAAAATGGCAGGAACTCCAATTGATGAAAACCTGTTAGCACATCCTGCTAGAAAATCAT  chr3

281  GCTGCTCTGCCTCGGTTTGAAATGTGAAGAAAGAAGAGTCTCCCTTTTATCACTTTGGTAGCCAGGAGCT  272-12L_T7
     GCTGCTCTGCCTCGGTTTGAAATGTGAAGAAAGAAGAGTCTCCCTTTTATCACTTTGGTAGCCAGGAGCT  chr3
```

```
351   ATTGGACAAATTACACATAAAAAATAATATTTAAGGGTCAGGTAATTATTCTTATAACTTGGGGGAACTAG   272-12L_T7
      ATTGGACAAATTACACATAAAAAATAATATTTAAGGGTCAGGTAATTATTCTTATAACTTGGGGGAACTAG   chr3

421   CTCAGTAATAGAGGAAACATTCTCCTATCTCCTCTTAATTCTGAAGTCTAAACTTCTTTGGTGCCAGCCT   272-12L_T7
      CTCAGTAATAGAGGAAACATTCTCCTATCTCCTCTTAATTCTGAAGTCTAAACTTCTTTGGTGCCAGCCT   chr3

491   GCTTTCAAATCAGATGACACTGCTGCTGGCTGTCATTCCATGATGTTAGAGGAAGTGTTGAGCNTT-AAT   272-12L_T7
      GCTTTCAAATCAGATGACACTGCTGCTGGCTGTCATTCCATGATGTTAGAGGAAGTGTTGAGCATTTAAT   chr3

560   GAATTC   272-12L_T7
      GAATTC   chr3
```

>275-12L_SP6 vs. chr10: 26,997,857-26,998,175 (-), 320 bp overlap

```
  1   TATAGCAGAGGATGGCCTAGTCAGTGATCAATGGGAAGAGAAGCCCTTGGTCTTGTGAAGGCTCTATGCC   275-12L_SP6
      TATAGCAGAGGATGGCCTAGTCAGTGATCAATGGGAAGAGAAGCCCTTGGTCTTGTGAAGGCTCTATGCC   chr10

 71   CCAGTATAGGGGAATTCCAGGGCCAGGAAGCAGGAGTGGGTGGGTTGGGGAGCAGGGGAAGGGGGGGAGG   275-12L_SP6
      CCAGTATAGGGGAATTCCAGGGCCAGGAAGCAGGAGTGGGTGGGTTGGGGAGCAGGGGAAGGGGGG-AGG   chr10

141   GGATAGGGAATTTTTGGAGAGGAAACTAGGAAAGGGGATAACATTTGAAATGAAAACAAAGAAAATATCT   275-12L_SP6
      GGATAGGGAATTTTTGGAGAGGAAACTAGGAAAGGGGATAACATTTGAAATGAAAACAAAGAAAATATCT   chr10

211   AATAAAAATAATTTACATGAGTATAAAAATATGGACATCCTGACTATACTTTTTCCTATTCATCAATTTT   275-12L_SP6
      AATAAAAATAATTTACATGAGTATAAAAATATGGACATCCTGAGTACACTTTTTCCTATTCATCAATTTT   chr10

281   TCAAGTCAAATCATGGCATTTTCACAAAATAACCTTCAAA   275-12L_SP6
      TCAAGTCAAACCATGGCATTTTCACAAAATAACCTTCAAA   chr10
```

>275-12L_T7 vs. chr10: 26,884,138-26,884,774 (+), 634 bp overlap

```
  1   GAATTCTTCAATATAGAGCATACATAGGTCATTTTTCCTTCCCATTTGAGAGAGTCATCCAGGCAAAGAA   275-12L_T7
      GAATTCTTCAATATAGACCACACATAGGTCATTTT-CTTTCCCATTTGAGAGAGTCATCCAGGCAAAGAA   chr10

 71   AAGCAATGTGACAGTTGCTAGAGAATTAAAAGAACTGCTAAAGCATTGTCCATCTTCAGGACAATGCTTG   275-12L_T7
      AAGCAATGTGACAGTTGCTAGAGAATTAAAAGAACTGCTAAAGCATTGTCCATCTTCAGGACAATGCTTG   chr10

141   TGTAGAATGTGTGCTATTGTCACCTAATATGCCAGACTAAACTGGAAGCATATTTGGAGTTCCAGTATCT   275-12L_T7
      TGTAGAATGTGTGCTATTGTCACCTAATATGCCAGACTAAACTGGAAGCATATTTGGAGTTCCAGTATCT   chr10

211   ATATAGAAATAGTTATGTTGTGTGCACTAACATGCTTTAGGGGGGCAGAAATGTAGGATTCAAAAGGTAG   275-12L_T7
      ATATAGAAATAGTTATGTTCTGTGCACTAACATGCTTTAGGGGGGCAGAAATGTAGGATTCAAAAGGTAG   chr10

281   GATTTTTAGGGGAATTTAGAGAAAACACTTTTCAAGGAGGAGGAGGAGG---TAGTGAAGGAAG-TAATA   275-12L_T7
      GATTTTTAGGGGAATTTAGAGAAAACACTTTTCAAGGAGGAGGAGGAGGAGGTAGTGAAGGAAGATAATA   chr10

347   ATCTATCACCTGAAAGAAGGAACCTAGTGGACTATTTCATAAGGTGGGAACAGAATAACTTCTCTGTGGA   275-12L_T7
      ATCTATCACCTGAAAGAAGGAACCTAGTGGACTATTTCATAAGGTGGGAACAGAATAACTTCTCTGTGGA   chr10

417   ACTTACAGCTTGTCTCAAAGCTATGTGAAACTCATTCCTAGCCTAGATGAGGAAAATCAGCGGGAGACAG   275-12L_T7
      ACTTACAGCTTGTCTCAAAGCTATGTGAAACTCATTCCTAGCCTAGATGAGGAAAATCAATGGGAGACAG   chr10

487   TCACTGCCAGCGCCTTCGTATGGAGTTTACCTTTGCCTAGTATTTTGTTTTTACTTCAAACTCATTCATC   275-12L_T7
      TCACTGCCAGCGCCTTCGTATGGAGTTTACCTTTGCCTAGTATTTTGTTTTTACTTCAAACTCATTCATA   chr10

557   CCCTTCCAATCCTCCTTATCCCTACATGTTGATGGAAACAATTGCAGTGTTGGTCTCTGGCCTTTATCTG   275-12L_T7
      TCCTTCCAATCCTCCTTATCCCTACATGTTGATGGAAACAATTGCAGTGTTGGTCTCTGGCCTTTATCTG   chr10

627   TTCTTCAA   275-12L_T7
      TTCTTCAA   chr10
```

>275-24H_SP6 vs. chr14: 40,674,048-40,674,533 (+), 515 bp overlap

```
  1  GAATTCCAAGAGGCAACCAACACTGTGGGTGTAGGGTCCTTTAATGGCCAGACACACAAAATAACTCATG  275-24H_SP6
     GAATTCCAAGAGGCAACAAACACTGTGGGTGTAGGGTCCTTTAATGGCCAGACACACAAAATAACTCATG  chr14

 71  TTACAGGACCCCCAAAAGGTCCTGGCAATTGAAGAGGGTGAGGATGGCAGGGGCAGGAAGACATTTTCTT  275-24H_SP6
     TTACAGGACCCCCAAAAGGTCCTGGCAATTGAAGAGGGTGAGGATGGCAGGGGCAGGAAGACATTTTCTT  chr14

141  CTTTCAGCCTTGAAAGTCCCTTGGAGGCTTAGAGGGTCTAATTATCTTTTGATTTTTCTTCTTCTTCTTC  275-24H_SP6
     CTTTCAGCCTTGAAAGTCCCTTGGAGGCTTAGAGGGTCTAATTATCTTTTGATTTTTCTTCTTCT---TC  chr14

211  ATACCAGACAGCCTTGGAAGACAGTGTGTCACTTAGACATGGCTAGTGTGTGGTGTGGATGGTACAGCCT  275-24H_SP6
     ATACCAGACAGCCTTGGAAGACAGTGTGTCACTTAGACATGGCTAGTGTGTGGTGTGGATGGTACAGCCT  chr14

281  GGGTGATGCTGGAGTGGTTAAGAATTCACATTTCAGACTCACCTACCTTCTTGGAGCCTAGTCTTTGGCT  275-24H_SP6
     GGGTGATGCTGGAGTGGTTAAGAATTCACATTTCAGACTCACCTACCTTCTTGGAGCCTAGTCTTTGGCT  chr14

351  GGGTCTTCTAAGTGGGTCCAGTTAGATTTCAAAGGTACTGGCTCTTCTGTTTGCAGCAGACCTCTCTGAG  275-24H_SP6
     GGGTCTTCTAAGTGGGTCCAGTTAGATTTCAAAGGTACTGGCTCTTCTGTTTGCAGCAGACCTCTCTGAG  chr14

421  GGATGGGGCTGTTGACCCTTGATAGTCCTAGGTTTCCTCTCTCCCTAGTCGTCTTTCTTTCTTTCTTTCT  275-24H_SP6
     GGATGGGGCTGTTGACCCTTGATAGTCCTAGGTTTCCTCTCTCTCTAGT--TCTTTCTTTCTTTCTTTCT  chr14

491  TCTTCTTCTTCTTCTTCTTCTTCTT  275-24H_SP6
     TTCTTTCTTTCTTTCTTTCTTTCTT  chr14
```

>275-24H_T7 vs. chr14: 40,833,328-40,833,901 (-), 616 bp overlap

```
  1  GTCACAGTTCTCTAGGGGTAATGGGACCAGATGGGAGGGAGAGGGGAGGAGAGCTGGTTGTTAGAAGCTG  275-24H_T7
     GTCACAGTTCTCTAGGGGTAATGGGACCAGATGGGAGGGAGAAGGGAGGAGAGCTGGTTGTTAGAAGCTG  chr14

 71  GTAGTTCGGGACCTGAAATGGTTTGGGCATCAGATGAACGCTGTGAGAATATCACCAGCCATTCTAAAGC  275-24H_T7
     GTAGTTCGGGACCTGAAATGGTTTGGGCATCAGATGAACGCTGTGAGAATATCACCAGCCATTCTAAAGC  chr14

141  CCAACACCGCCTTAGCACTGCCTGTGGCAGCTCAGGTCCTCAGGGTCCCCAGCAGAGGGTCCCCAGCAGA  275-24H_T7
     CCAACACCGCCTTAGCACTGCCTGTGGCAGCTCAGGTCCTCAGGGTCCC---------------------  chr14

211  GGGTCCCCAGCAGAGGGTCCCCAGCAGAGACCACAGAGTGTCACACCTGGCTTGGGAAGTTTCAGACTCT  275-24H_T7
     ---------------------CAGCAGAGACCACAGAGTGTCACACCTGGCTTGGGAAGTTTCAGACTCT  chr14

281  CACCTCTATCTGCATCTTGTTGGCAAGTAAGTCATAAGCCATGAAGGTCAGCCCACATTTAGGGAAGAGG  275-24H_T7
     CACCTCTATCTGCATCTTGTTGGCAAGTAAGTCATAAGCCATGAAGGTCAGCCCACATTTAGGGAAGAGG  chr14

351  AAGTTAGACAGACTCTACTTTTCAGTAGAGCAGTGATGAGGGATTTGTGTCCCTCGTTAACCCACCAGAC  275-24H_T7
     AAGTTAGACAGACTCTACTTTTCAGTAGAGCAGTGATGAGGGATTTGTGTCCCTCGTTAACCCACCAGAC  chr14

421  ATGTTCCTTGATAGGAAAGCCCCTGAGAGACCTTGAAGAGGAAGGGGTGGAAGGACGTTGGGCATCCCTG  275-24H_T7
     ATGTTCCTTGATAGGAAAGCCCCTGAGAGACCTTGAAGAGGAAGGGGTGGAAGGACGTTGGGCATCCCTG  chr14

491  ATCAGTGACGAAGTGCACTTTCCCATCTGAGACAGAAACCAAATGATGATAGGCTGAAGCAGTCAGAGCA  275-24H_T7
     ATCAGTGACGAAGTGCACTTTCCCATCTGAGACAGAAACCAAATGATGATAGGCTGAAGCAGTCAGAGCA  chr14

561  TGGTAGGTCTGGGAAACTGGAGTGGGTGATGGATGCACAGTGCTTTCCTAGAATTC  275-24H_T7
     TGGTAGGTCTGGGAAACTGGAGTGGGTGATGGATGCACAGTGCTTTCCTAGAATTC  chr14
```

>279-5I_SP6 vs. chr17: 12,057,702-12,058,065 (+), 365 bp overlap

```
  1  GAATTCATTTGGAACCTGGTGAATATATAGAAGTGTCTGGGAAGGCAAAGGCTTCTAGGAATCTTCCAAG  279-5I_SP6
     GAATTCATTTGGAACCTGGTGAATATATAGAAGTGTCTGGGAAGGCAAAGGCTTCTAGGAATCTTCCAAG  chr17

 71  TGGCCAGTCCTCCTTCCTCTCTCCACTCAGCTCCTGACACGGCATCCCTGTCAGGTATGTGGGGATTTGC  279-5I_SP6
     TGGCCAGTCCTCCTTCCTCTCTCCACTCAGCTCCTGACACGGCATCCCTGTCAGGTATGTGGGGATTTGC  chr17

141  CTGTTTGGACCTGAAGGTAAATCGTGTTTTCTTTGGCCTTTGATTTCCTGTAGCAACCATAAATAAAAAT  279-5I_SP6
     CTGTTTGGACCTGAAGGTAAATCGTGTTTTCTTTGGCCTTTGATTTCCTGTAGCAACCATAAATAAAAAT  chr17
```

```
211  TTAAATTAATGAAAAATCAACACCTTGCTTGTCTGATTCTTGAGACGGGGTCTTGTAATACAGCCCTTGC  279-5I_SP6
     TTAAATTAATGAAAAATCAACACCTTGCTTGTCTGATTCTTGAGACGGGGTCTTGTAATACAGCCCTTGC  chr17

281  CAGGCTGGGTGGCAGTGTTGCACAACCTTTAATCCCAGCACTTGGGAGGCAGAGGCAGGTGGATTTCTGA  279-5I_SP6
     CAGGCTGGGTGGCAGTGGTGCACA-CCTTTAATCCCAGCACTTGGGAGGCAGAGGCAGGTGGATTTCTGA  chr17

351  GTTCAAGGCCAGCCT  279-5I_SP6
     GTTCAAGGCCAGCCT  chr17
```

>279-5I_T7 vs. chr17: 12,182,179-12,182,701 (-), 523 bp overlap

```
  1  CCTAGAGCACTGGATCATGCTATAGCCCATGGGTCGAATCAGGTCACACAGCTATTTTGTAAATATTTTT  279-5I_T7
     CCTAGAGCACTGGATCATGCTATAGCCCATGGGTCGAATCAGGTCACACAGCTATTTTGTAAATATTTTT  chr17

 71  ACTGGGATACAGCCATCCCCATTGCTTATGTGTTGGACTGGCTGATTTTGCCCACAAGAGCAGAGATAAT  279-5I_T7
     ACTGGGATACAGCCATCCCCATTGCTTATGTGTCGGACTGGCTGATTTTGCCCACAAGAGCAGAGATAAT  chr17

141  AGAAACCATGGCCTGAAGAGCCTAAAGTATCTACATTTAACCATGACCCTCACCAAGAAAACTCACCCAA  279-5I_T7
     AGAAACCATGGCCTGAAGAGCCTAAAGTATCTACATTTAACCATGACCCTCACCAAGAAAACTCACCCAA  chr17

211  CACCTCTTTTAGGTTCCCAGTTCCCAGGAGTGCTGCCTTTAAACCAGCAGTTTGGGTCTCATCTGCCAGC  279-5I_T7
     CACCTCTTTTAGGTTCCCAGTTCCCAGGAGTGCTGCCTTTAAACCAGCAGTTTGGGTCTCATCTGCCAGC  chr17

281  CTATTAGAACTCTCAGTTCTAATACAAGCTATAACAGTTTAATTTGTTGGGCATAGTAATTCCAGCTCTG  279-5I_T7
     CTATTAGAACTCTCAGTTCTAATACAAGCTATAACAGTTTAATTTGTTGGGCATAGTAATTCCAGCTCTG  chr17

351  GAACAAGTAGGAGGATCAGACATTCAAAATCATCTTTAGCTACGAGTATTTTCCAGGCCAGCCTGCACTA  279-5I_T7
     GAACAAGTAGGAGGATCAGACATTCAAAATCATCTTTAGCTACGAGTATTTTCCAGGCCAGCCTGCACTA  chr17

421  TATGAGACCTGATATTTAAAGTTAAAATGTTTTAAATATATATCAGAAACATCACACATGGTGGTGCATC  279-5I_T7
     TATGAGACCTGATATTTAAAGTTAAAATGTTTTAAATATATATCAGAAACATCACACATGGTGGTGCATC  chr17

491  ACTATAATCACAACACCTGGGAGGCACAGGCTG  279-5I_T7
     ACTATAATCACAACACCTGGGAGGCACAGGCTG  chr17
```

>282-5D_SP6 vs. chr17: 12,172,619-12,172,970 (-), 352 bp overlap

```
  1  CAAAACACAGAAGATAAAAATAAATTTAAAAACAACAACAACAACAAACAAACCACCTCAGCATCCAGGA  282-5D_SP6
     CAAAACACAGAAGATAAAAATAAATTTAAAAACAACAACAACAACAAACAAACCACCTCAGCATCCAGGA  chr17

 71  TGGTGAATACAATGACAGTTAGGGGGAAATGAACCTTCCCCGTCCCCTCACAGATCCTAGAGTTAAACAA  282-5D_SP6
     TGGTGAATACAATGACAGTTAGGGGGAAATGAACCTTCCCCGTCCCCTCACAGATCCTAGAGTTAAACAA  chr17

141  TAAGGAAATCTGTGTCCTTGGGGTGCCCAGTGATTCCCAAAGAGGAGAGATTTTGTCTGGTTGGAGTTTC  282-5D_SP6
     TAAGGAAATCTGTGTCCTTGGGGTGCCCAGTGATTCCCAAAGAGGAGAGATTTTGTCTGGTTGGAGTTTC  chr17

211  ATCCGAAATGTCTAGGGACATTTTCCTTTGTGTCACCAAGGTAAGGGCATATGGTGAAGAGCCCTGGACC  282-5D_SP6
     ATCCGAAATGTCTAGGGACATTTTCCTTTGTGTCACCAAGGTAAGGGCATATGGTGAAGAGCCCTGGACC  chr17

281  TACTGGGTATGGGCCAGGAACATTGCCTAACACCCAACAATGCACAGGAAGGCTCCTCCAGGGATGGAAT  282-5D_SP6
     TACTGGGTATGGGCCAGGAACATTGCCTAACACCCAACAATGCACAGGAAGGCTCCTCCAGGGATGGAAT  chr17

351  TC  282-5D_SP6
     TC  chr17
```

>282-5D_T7 vs. chr17: 12,028,446-12,028,807 (+), 349 bp overlap

```
  1  GAATTCCACATCTCATAGCAAGGAATCAAAGCAAACTACATCAAAGCATTAGACATCAAACAGAGACAAG  282-5D_T7
     GAATTCCACATCTCATAGCAAGGAATCAAAGCAAACTACATCAAAGCATTAGACATCAAACAGAGACAAG  chr17
```

```
 71  AAATTAGTGGAAATGCCATAGGAGGATTGGTTATAATTCACAGCTTCATAATGGGCAAGGTCAGTCTAAA  282-5D_T7
     AAATTAGTGGAAATGCCATAGGAGGATTGGTTATAATTCACAGCTTCATAATGGGCAAGGTCAGTCTAAA  chr17

141  TGTGACAAAAAAAAAAA-------------CAGAAACAACAACAAAAACAACAACAACAACAAAAATGGT  282-5D_T7
     TGTGACAAAAAAAAAAAAAAAAAAAAAAAACAGAAACAACAACAAAAACAACAACAACAACAAAAATGGT  chr17

198  CCAGGGCCTATGGGAGAGGGATTAGTCTGTATGCATTCACCACAGAAATACAGAGTTGAATTGAAACGCT  282-5D_T7
     CCAGGGCCTGTGGGAGAGGGATTAGTCTGTATGCATTCACCACAGAAATACAGAGTTGAATTGAAACGCT  chr17

268  GTCACCACTGGTAAACCTGGATAAAGTGTTTAGTGCTATACAAAGGGCTGGCTTTCTGAATGTATGTGGA  282-5D_T7
     GTCACCACTGGTAAACCTGGATAAAGTGTTTAGTGCTATACAAAGGGCTGGCTTTCTGAATGTATGTGGA  chr17

338  AAGCTCTAACAA  282-5D_T7
     AAGCTCTAACAA  chr17
```

>293-21P_SP6 vs. chrX: 44,270,572-44,271,061 (-), 491 bp overlap

```
  1  CAACCAGCCACCTTCCTGGCGAGAGCCACAGAGCTTCTGAGGCGGCGACATCTTCGACTCCAGACAACCG  293-21P_SP6
     CAACCAGCCACCTTCCTGGCGAGAGCCACAGAGCTTCTGAGGCGGCGACATCTTCGACTCCAGACAACCG  chrX

 71  GCCNNCCTTCCTGGCCAAAGCAACACAGCTTCTGGGAAAGATCCTGTTTTGGGCCTTCACCTTCAGCCAG  293-21P_SP6
     GCCA-CCTTCCTGGCCAAAGCAACACAGCTTCTGGGAAAGATCCTGTTTTGGGCCTTCACCTTCAGCCAG  chrX

141  GAGGAGGTCCAAACACCAGATAACTGTACACCTTCCCCGAAAGAGGAGAGCTTGCCTGCAGAGACTGCTC  293-21P_SP6
     GAGGAGGTCCAAACACCAGATAACTGTACACCTTCCCCGAAAGAGGAGAGCTTGCCTGCAGAGACTGCTC  chrX

211  TGACCACTGAAACTCAGAGGAGAGAGCTTGTCTCCCACGCCTGCTGATTGAGGGTAACAAAATCAACAGA  293-21P_SP6
     TGACCACTGAAACTCAGAGGAGAGAGCTTGTCTCCCACGCCTGCTGATTGAGGGTAACAAAATCAACAGA  chrX

281  GGAACAATCTCTTAACAAAGACAACTATAACAACTAACTCCAGAGATTGCCAGATGGCGAAAGGTAAACG  293-21P_SP6
     GGAACAATCTCTTAACAAAGACAACTATAACAACTAACTCCAGAGATTGCCAGATGGCGAAAGGTAAACG  chrX

351  TAAGAATCCTACTAACAGAAACCAGGACCACTCACCATCATCAGAACCCAGAACGCCCACTTCGCCCAGT  293-21P_SP6
     TAAGAATCCTACTAACAGAAACCAGGACCACTCACCATCATCAGAACCCAGAACGCCCACTTCGCCCAGT  chrX

421  CCAGGACACCCTAACACACCTGAAAAGGTAGACCTGGATTTAAAAGCATATCTCATGATGATGGTAGAGG  293-21P_SP6
     CCAGGACACCCTAACACACCTGAAAAGGTAGACCTGGATTTAAAAGCATATCTCATGATGATGGTAGAGG  chrX

491  A   293-21P_SP6
     A   chrX
```

>293-21P_T7 vs. chrX: 44,086,068-44,086,651 (+), 586 bp overlap

```
  1  AATGCAGTAAACTGCAATGCCTGATTACTTTCACTATCTCTCTGTCAACACTGGAGGCTTTTTGTCTGAA  293-21P_T7
     AATGCAGTAAACTGCAATGCCTGATTACTTTCACTATCTCTCTGTCAACACTGGAGGCTTTTTGTCTGAA  chrX

 71  TGAATAGCATTCTTACTAAATTCAACGATTTCCTAAAATATTGGAACACTGGTGGAGGCCAATCTAACTT  293-21P_T7
     TGAATAGCATTCTTACTAAATTCAACGATTTCCTAAAATATTGGAACACTGGTGGAGGCCAATCTAACTT  chrX

141  ATCTATTTGTCAAATCATTCCTTTGAAGCACTTATAATAAAACAATACTGAAAGAAAACCCTTCACTAGA  293-21P_T7
     ATCTATTTGTCAAATCATTCCTTTGAAGCACTTATAATAAAACAATACTGAAAGAAAACCCTTCACTAGA  chrX

211  CTATCGCAAGGACATATCTGGGCCACATTGTGTTATGGAATGCCAAAAAGCACCAGGAGACCAGTTTCTG  293-21P_T7
     CTATCGCAAGGACATATCTGGGCCACATTGTGTTATGGAATGCCAAAAAGCACCAGGAGACCAGTTTCTG  chrX

281  TGAAACTTTTTGCCTCAGGGCTGCGGCCAAGCTTTTGGACTTGTCACGCATACTGACTGGAGTGGGTGTG  293-21P_T7
     TGAAACTTTTTGCCTCAGGGCTGCGGCCAAGCTTTTGGACTTGTCACGCATACTGACTGGAGTGGATGTG  chrX

351  GCACATACTACACACACACACACACACACACACACACACACACACACACACACACACACACACAGAGTCT  293-21P_T7
     GCACATACTACACACACAAACACACACACACACACACACACACACACACACACACACACACACAG--TCT  chrX

421  AGGGAGAAGGTGGATTATACAATAGACTAGGATTGTAACTATGGCAAGGGCATAAAGCCCTTGCCCCTGG  293-21P_T7
     AGGGAGAAGGTGGATTATACAATAGACTAGGATTGTAACTATGGCAAGGGCATAAAGCCCTTGCCCCTGG  chrX
```

```
491  CTTCTAAGAATAAGCTGACCTTAGGTAGGGCTGATTTTTATCACAGGTGTGTGTGTGTGTGTGTGTGT  293-21P_T7
     CTTCTAAGAATAAGCTGACCTTAGGTAGGGCTGATTTTTATCACAGGTGTGTGTGTGTGTGTGTGTGT  chrX

561  GTGTGTGTGTGTGTGTGTGTGTGTGTGT  293-21P_T7
     GTGTGTGTGTGTGTGTGTGTGTGTGTGT  chrX
```

>293-21P-B_SP6 vs. chr17: 38,817,382-38,817,789 (-), 408 bp overlap

```
  1  CCCAACCCACCCACTCCCACTTCCTGGCCCTGGCATTCCTCTGTACTGGGGTTTGTGATTTATTTATTAA  293-21P-B_SP6
     CCCAACCCACCCACTCCCACTTCCTGGCCCTGGCATTCCTCTGTACTGGGGTTTGTGATTTATTTATTAA  chr17

 71  GTAAAGGACTTCACTACTTAATACCCAGCTATTAAAAAAAAAGAAAACCAAACCAGTGTCAATAGACCAA  293-21P-B_SP6
     GTAAAGGACTTCACTACTTAATACCCAGCTATTAAAAAAAAAGAAAACCAAACCAGTGTCAATAGACCAA  chr17

141  GAAGGCTGCTGCTTCTGGCCTCAGAGTAACTTAACCTCAAATGAGCTTCTAGTCAAGTTCACCACTCACT  293-21P-B_SP6
     GAAGGCTGCTGCTTCTGGCCTCAGAGTAACTTAACCTCAAATGAGCTTCTAGTCAAGTTCACCACTCACT  chr17

211  TCCCACCAGGAACTAAGAGCAGTTTCTGAGCCTGAATTTGTTGCTGTTTTAAAGGTTATTGTGGGAACCT  293-21P-B_SP6
     TCCCACCAGGAACTAAGAGCAGTTTCTGAGCCTGAATTTGTTGCTGTTTTAAAGGTTATTGTGGGAACCT  chr17

281  GAATTAAAGGAGGTGGCATCTTCATTTTTGTATCAAAGATAAAGGTTATTTGGGAAATTATTAGGGATTT  293-21P-B_SP6
     GAATTAAAGGAGGTGGCATCTTCATTTTTGTATCAAAGATAAAGGTTATTTGGGAAATTATTAGGGATTT  chr17

351  TACATATGTATGAAGTTTGGTGCTTTTGTAAACAAATTGTTCAATTCCTTAGGAATTC  293-21P-B_SP6
     TACATATGTATGAAGTTTGGTGCTTTTGTAAACAAATTGTTCAATTCCTTAGGAATTC  chr17
```

>297-2I_SP6 vs. chr17: 14,439,105-14,439,480 (-), 376 bp overlap

```
  1  TTATCTGGAAAATAGTTGCTGCAAGGGGCAGTGACTGGATACATTTTACGGCTAAGTGTGAACAATCCAG  297-2I_SP6
     TTATCTGGAAAATAGTTGCTGCAAGGGGCAGTGACTGGATACATTTTACGGCTAAGTGTGAACAATCCAG  chr17

 71  GAAGATTTTCGGAGAAGTGGATTGGGTGACTAAATAAGAGATACGGCATTTATTAAGATATGTTTAAGCA  297-2I_SP6
     GAAGATTTTCGGAGAAGTGGATTGGGTGACTAAATAAGAGATACGGCATTTATTAAGATATGTTTAAGCA  chr17

141  GATGAGCAGGGTAAGGAAAAAGGAGGAGGTTCACTTTTATAAGCATTGAGTTTATTGAATGAAGTATAAA  297-2I_SP6
     GATGAGCAGGGTAAGGAAAAAGGAGGAGGTTCACTTTTATAAGCACTGAGTTTACTGAATGAAGTATAAA  chr17

211  ATTCTACCTAAGGCCCAGGATTGTCAGCCAGTGTAACTTACGATGTAACAGTGACAGAATTCGAAGCCAT  297-2I_SP6
     ATTCTACCTAAGGCCCAGGATTGTCAGCCAGTGTAACTTACGATGTAACAGTGACAGAATTCGAAGCCAT  chr17

281  GCATCTCAGAAGAAACTCAGGGTCCCAAGAAAGCCAACATTGTTCAAACACACCTCTACTCTCAGCACTA  297-2I_SP6
     GCATCTCAGAAGAAACTCAGGGTCCCAAGAAAGCCAACATTGTTCAAACACACCTCTACTCTCAGCACTA  chr17

351  GGTAGGAGAAGGCAGGATGATCAGGA  297-2I_SP6
     GGTAGGAGAAGGCAGGATGATCAGGA  chr17
```

>297-2I_T7 vs. chr17: 14,291,556-14,292,032 (+), 513 bp overlap

```
  1  GAATTCCAGGTCAACTAAAGCTTCATAGAGAGACCTTGTCACAAAATTCATTCATTCATTCATTCATTCA  297-2I_T7
     GAATTCCAGGTCAACTAAAGCTTCATAGAGAGACCTTGTCACAAAATTCATTCATTCATTCAT-------  chr17

 71  TTCATTCATTCATTCATTCATTCATTCATAAACCCAAAGACCCTTAAATGTGGGCTTCAGTGGAAAAAGA  297-2I_T7
     -----------------------------AAACCCAAAGACCCTTAAATATGGGCTTCAGTGGAAAAAGA  chr17

141  TAAATTACATAAACAACACACTTAAGCAAAACTGAAGTCTAGCTGTATAGATCAATGTCTATCATGTCCA  297-2I_T7
     TAAATTACATAAACAACACACTTAAGCAAAACCGAAGTCTAGCTGTATAGATCAATGTCTATCATGTCCA  chr17

211  CTTGTCCTTTTACAATACACTCTTCTGGGCTCTAAAGGGCTCTCCAACTCTGCTGTCCCCAGCACACATG  297-2I_T7
     CTTGTCCTTTTACAATACACTCTTCTGGGCTCTAAAGGGCTCTCCAACTCTGCTGTCCCCAGCACACATG  chr17
```

```
281   GTGGTAGTACAGGCTCAGGTCAGATTCATCCCACACTTACCACTGTTCTTAGCAGATATCCCAGGTCCGA   297-2I_T7
      GTGGTAGTACAGGCTCAGGTCAGATTCATCCCACACTTACCACTGTTCTTAGCAGATATCCCAGGTCCGA   chr17

351   GCTGCTCCAATATCTGCTACAACTCATGTTCTGCCTTCACCAATAGCTTCTTGGGCCTCTCTGCAGGTAC   297-2I_T7
      GCTGCTCCAATATCTGCTACAACTCATGTTCTGCCTTCACCAATAGCTTCTTGGGCCTCTCTGCAGGTAC   chr17

421   CCCAACCCTGCAACAGGGTGCCAGTCCTCAGCTTCTCTTTATGACCCCTTCAATCAAGAGCTTTCCTGCC   297-2I_T7
      CCCAACCCTGCAACAGGGTGCCAGTCCTCAGCTTCTCTTTATGACCCCTTCAATCAAGAGCTTTCCTGCC   chr17

491   TTTAAAACCAGTAACAGGTGGGT   297-2I_T7
      TTTAAAACCAGTAACAGGTGGGT   chr17
```

>300-19K_T7 vs. chr1: 31,429,439-31,430,003 (+), 562 bp overlap

```
  1   GAATTCTAGAAGCA---GGAGAGCTATGGTTTGGAGAGATTGAGCGGTTTCCGATTGTGAAAGATCAGGA   300-19K_T7
      GAATTCTAGAAGCAAGAGGAGAGCTATGGTTTGGAGAGATTGAGCGGTTTCCGATTGTGAAAGATCAGGA   chr1

 68   AGGTGAACATGTAATTTGACTACACTTTTAAACCGGGGGCCTCTAGTATCTGATTAGAGTTAGAAGAGGC   300-19K_T7
      AGGTGAACATGTAATTTGACTACACTTTTAAACCGGGGGCCTCTAATATCTGATTAGAGTTAGAAGAGGC   chr1

138   CATCCAGGGGCAGCAGGCTTTGTGACAGTGAAGTGGGGGAGGCTGTGAAGCACACTGCCAAATCTGACGC   300-19K_T7
      CATCCAGGGGCAGCAGGCTTTGTGACAGTGAAGTGGGGGAGGCTGTGAAGCACACTGCCAAATCTGACGC   chr1

208   TGCACAAGCTAACGCTGCCTCAAGGGCACTGTGACAGAAACTTCACTGTCAGGTAAGTATTTGGGTGGAG   300-19K_T7
      TGCACAAGCTAACGCTGCCTCAAGGGCACTGTGACAGAAACTTCACTGTCAGGTAAGTATTTGGGTGGAG   chr1

278   AACTCTTTACAAGTTCCCTCACTACAGGCCATGGCTCTCTGTTGTCATGTCTACTCTTACCAAAGTATCT   300-19K_T7
      AACTCTTTACAAGTTCCCTCACTACAGGCCATGGCTCTCTGTTGTCATGTCTACTCTTACCAAAGTATCT   chr1

348   TCAATTTCTAAAACAGTGGGCAAGGAAAAGGAGTGCTTGCCCGTCCTCCCATCATTTGGGAGGTGGAGGA   300-19K_T7
      TCAATTTCTAAAACAGTGGGCAAGGAAAAGGAGTGCTTGCCCGTCCTCCCATCATTTGGGAGGTGGAGGA   chr1

418   AGGAGGATCACAAATTCAAGGTCACCCTTGGCCTTGTAATGAGTTTGAAGGCTGTCTAGGCTACATGAAA   300-19K_T7
      AGGAGGATCACAAATTCAAGGTCACCCTTGGCCTTGTAATGAGTTTGAAGGCTGTCTAGGCTACATGAAA   chr1

488   CCATGTCTTTAAAAGAAATACCAGACTTTGTTCTTCGTTGTATTCTCACCAGTTAAAGTGTATTTGGTGT   300-19K_T7
      CCATGTCTTTAAAAGAAACACCAGACTTTGTTCTTCGTTGTATTCTCACCAGTTAAAGTGTATCTGGTGT   chr1

558   TAGCA   300-19K_T7
      TAGCA   chr1
```

>301-19K_SP6 vs. chr8: 35,545,676-35,546,221 (+), 545 bp overlap

```
  1   GAATTCACTTTGTAGACCAGGCTGGCCTCGAACTCAGAAATCTGCCTGCTTTTGCCTCCCAAGTGCTCGG   301-19K_SP6
      GAATTCACTTTGTAGACCAGGCTGGCCTCGAACTCAGAAATCTGCCTGCTTTTGCCTCCCAAGTGCTCGG   chr8

 71   ATTAAAGGTGTGAGCCACCACCGCCCGGCTCTTTTTTTTTTTTTT-AATTAAAACAAAACAAAACAAAA   301-19K_SP6
      ATTAAAGGTGTGAGCCACCACCGCCCGGCTCTTTTTTTTTTTTTTTAATTAAAACAAAACAAAACAAAA   chr8

140   CAAAAAAACCCACTTTTACATTTCATGAATGTCTCAGGTCATGGACATCAAACAAGCAGTGAACAGTTCT   301-19K_SP6
      CAAAAAAACCCACTTTTACATTTCATGAATGTCTCAGGTCATGGACATCAAACAAGCAGTGAACAGTTCT   chr8

210   GGAAGAGTTATGTGACATTTCCTCAAAGCTTTCTGAAACTGCTCCTTCAATAGCCTTCAGACTATCGAAT   301-19K_SP6
      GGAAGAGTTATGTGACATTTCCTCAAAGCTTTCTGAAACTGCTCCTTCAATAGCCTTCAGACTATCGAAT   chr8

280   TCAGACTTGGGAGTGACGTAACAAATAAATCAGCCTTAATCACAGATCACTTGGCACTCAATGCTATTTC   301-19K_SP6
      TCAGACTTGGGAGTGACGTAACAAATAAATCAGCCTTAATCACAGATCACTTGGCACTCAATGCTATTTC   chr8

350   CCTATCCACTTGTTAGCTGTTTCTAGCCAGAATATCTATATTCGATGTGGCAGAAATCTAGCACCATGGT   301-19K_SP6
      CCTATCCACTTGTTAGCTGTTTCTAGCCAGAATATCTATATTCGATGTGGCAGAAATCTAGCACCATGGT   chr8

420   GGTGCACAGTCACTTGAAAAGGCGTTCCCTTTCTGAGCTCAGTCACTACCCAGTGAAGCACCCTGTTGGG   301-19K_SP6
      GGTGCACAGTCACTTGAAAAGGCGTTCCCTTTCTGAGCTCAGTCACTACCCAGTGAAGCACCCTGTTGGG   chr8
```

```
490  ACAAACATGACTGTGCTTTGAAATCGCTCATCACTGTTCCCGACATCTACCTCTGG  301-19K_SP6
     ACAAACATGACTGTGCTTTGAAATCGCTCATCACTGTTCCCGACATCTACCTCTGG  chr8
```

>301-19K_T7 vs. chr8: 35,675,856-35,676,404 (-), 574 bp overlap

```
  1  TCTCTCTCTCTCTCTTCTTCTTCTTCTTCAATTTTCAATATTAAGAGAAAAGAAAGAACAATATGTGT  301-19K_T7
     TTTCTTTCTTTCTTTCTTTCTTTCTTTCTTCAATTTTCAATATTAAGAGAAAAGAAAGAACAATATGTGT  chr8

 71  TTCTACCACTGTTATATTTGTTTCTGTTTGTGAAGACTTAAGCTTACAGATAGCTTGTAATTGTCTTGCT  301-19K_T7
     TTCTACCACTGTTATATTTGTTTCTGTTTGTGAAGACTTAAGCTTACAGATAGCTTGTAATTGTCTTGCT  chr8

141  CAGAGATTATGAGAATTTGGGACTTGGAACTCAATGGAAATGAAGAAAGACAAGACAGACAGTACAATAG  301-19K_T7
     CAGAGATTATGAGAATTTGGGACTTGGAACTCAATGGAAATGAAGAAAGACAAGACAGACAGTACAATAG  chr8

211  AAAGACTGAAAAAAGAATTCGTTCTTTGAAAAGTCAAATGACATTCACAAACCCTTTGCCACACTAACCA  301-19K_T7
     AAAGACTGAAAAAAGAATTCGTTCTTTGAAAAGTCAAATGACATTCACAAACCCTTTGCCACACTAACCA  chr8

281  AAAGGAAGAACAGACTGCAATCAATACAATTAGAGGTGAGAAGACGAACATTGGAACAGATACCAACAAA  301-19K_T7
     AAAGGAAGAACAGACTGCAATCAATACAATTAGAGGTGAGAAGACGAACATTGGAACAGATACCAACAAA  chr8

351  ATCCAGGAAGAAGTCATTAGGAAAGACCTTAAAACTTTCTATTCCACTACACTGGAAAGTACAAAAGAAA  301-19K_T7
     ATCCAGGAAGAAGTCATTAGGAAAGACCTTAAAACTTTCTATTCCACTACACTGGAAAGTACAAAAGAAA  chr8

421  TAGACGAAATCGTGGGTGTTTTGGCTTACCAAAATTAAACCAAGATGAAATAAATGATGTAAACAGACCT  301-19K_T7
     TAGACGAAATCGTGGGTGTTTTGGCTTACCAAAATTAAACCAAGATGAAATAAATGATGTAAACAGACCT  chr8

491  GTAACAAGCAATGAGGTAGAACCAGAAGTTAAAAATCTCCTCACCAAAAGAAGTCTTGGTCTAAAAGGAT  301-19K_T7
     GTAACAAGCAATGAGGTAGAACCAGAAGTTAAAAATCTCCTCACCAAAAGAAGTCTTGGTCTAAAAGGAT  chr8

561  TTGGGACAGAATTC  301-19K_T7
     TTGGGACAGAATTC  chr8
```

>304-90_SP6 vs. chrX: 43,514,389-43,514,994 (+), 608 bp overlap

```
  1  AATTCAACACAAGTTTCTAATTCACCATGAATGGAACACAAGGTACTATGACTTAATTAATGCGCTTAAA  304-90_SP6
     AATTCAACACAAGTTTCTAATTCACCATGAATGGAACACAAGGTACTATGACTTAATTAATGCGCTTAAA  chrX

 71  TCCACTCTTAAACACTTGTACAGAAGTGAGTCATGAAGAGAAATACTGAGGAATTGTTCACACTAAGATT  304-90_SP6
     TCCACTCTTAAACACTTGTACAGAAGTGAGTCATGAAGAGAAATACTGAGGAATTGTTCACACTAAGATT  chrX

141  TATTGCTGCCTAGATTCCTCCTGGAGAGGATTCTATTGCTCTCATCTTACAGATGGGAAAATGGAGGCAA  304-90_SP6
     TATTGCTACCTAGATTCCTCCTGGAGAGGATTCTATTGCTCTCATCTTACAGGTGGGAAAATGGAGGCAA  chrX

211  GGAAGGTGACTTCCACAACTTCACATAGCTAAGTAGGTTATAGAGCTGAGGTCCAAGCCAGGAAGTCTGA  304-90_SP6
     GGAAGGTGACTTGCACAACTTCACATAGCTAAGTAGGTTATAGAGCTGAGGTCCAAGCCAGGCAGTCTGA  chrX

281  CTCCATGGCCAGCCCTTTAAACCATATCAAAGCACTCGGTCATTCGTATGGCAAAGATCACATTTTTTAA  304-90_SP6
     CTCCATGGCCAGCCCTTTAAACCATATCAAAGCACTCGGTCATTCGTATGGCAAAGATCACATTTTTTAA  chrX

351  GTTATTCTAGCTGTCTGCATGACATAGCATTGCTCTGTCCAATTCCCCGATAGTAACGAATATGGCTTTT  304-90_SP6
     GTTATTCTAGCTGTCTGCATGACATAGCATTGCTCTGTCCAATTCCCCGATAGTAACGAATATGGCTTTT  chrX

421  CTGTTTTGTGCCTTTTATTTTGTCATCAGAATCTCAAGTACTGTTGTGCTTCAAGGACTCCTAGTTTGAA  304-90_SP6
     CTGTTTTGTGCCTTTTATTTTGTCATCAGAATCTCAAGTACTGTTGTGCTTCAAGGACTCCTAGTTTGAA  chrX

491  GGATACTAAGGTGGCACAGTAATTTTAACATTAGTCTATCATCTGTATCTTGCTAGGAAGGAACTTCATC  304-90_SP6
     GGATACAAAGGTGGCACAGTAATTT-AACATTAGTCTATCATCTGTATCTTGCTAGGAAGGAACTTCATC  chrX

561  ACTCCCTTGTGAAGAATCAGAGTTCTGGCTACCTCTTTAGGTTTTGAA  304-90_SP6
     ACTACCTTGTGA-GAATCAGAGTTCTGTCTACCTCTTTAGGTTTTGAA  chrX
```

>304-90_T7 vs. chrX: 43,642,044-43,642,641 (-), 599 bp overlap

```
  1  CCCAACATAAATGGTGAGGTAATGATGGCAGTGTTGGCAGTGTAGTGTCTGTCTTAGAGGGGAGCCTGGT  304-90_T7
     CCCAACATAAATGGTGAGGTAATGATGGCAGTGTTGGCAGTGTAGTGTCTGTCTTAGAGGGGAGCCTGGT  chrX

 71  GGACCTATATGAAACTTTTCCCAGTCCTCAGCTCTGTAAACTTCCGCTTGATGACAACTAGGAGTGATTT  304-90_T7
     GGACCTATATGAAACTTTTCCCAGTCCTCAGCTCTGTAAACTTCCGCTTGATGACAACTAGGAGTGATTT  chrX

141  GTCAACAACTGTCCACAGCCATGGTAATTCTCTGAAATGCCTCAAATTCCATATTAGCCATAAACGTTCC  304-90_T7
     GTCAACAACTGTCCACAGCCATGGTAATTCTCTGAAATGCCTCAAATTCCATATTAGCCATAAACGTTCC  chrX

211  ATGTTGGTTATAGAATTAAAATAAATAGGTAGGCATCTGGCATAGCTGGCAAGACTGGGATATCGATGCA  304-90_T7
     ATGTTGGTTATAGAATTAAAATAAATAGGTAGGCGTCTGGCATAGCTGGCAAGATTTGGATATCAATGCA  chrX

281  ATCTATCGTATTTACCAGAGCAAGTGGAAGTCACTGTTAGATCTAAGAATAGTAGTGATCAATTTCAAGA  304-90_T7
     ATCTATCGTATTTACCAGAGCAAGTGGAAGTCACTGTTAGATCTAAGAATAGTAGTGATCAATTTCAAGA  chrX

351  TCATTTTTTATGAACCTTCAGCCCTATAGACAAACCGTTCAAGTAGAAGAATTGAGAATTGAGTATATAG  304-90_T7
     TCATTTTTTATGAACCTTTAGCCCTATAGACAAACCGTTCAAGTAGAGGAATTGAGAATTGAGTATATAG  chrX

421  AATCTACTACCAGTAAAGATTTAGGGCAAAGCACCCCCAGTACCCAGAAAGAAAACCATTAGTTCTATGT  304-90_T7
     AATCTACTACCAGTAAAGATTTAGGGCAAAGCACCCCCAGTACCCAGAAAGAAAACCATTAGTTCTATGT  chrX

491  TTTCTAGGGTGATAGGGCAAAGTAGATAATATGTAGGATTCACATTTTTTTTCTCCCCTAGCCTGAGAAA  304-90_T7
     TTTCTAGGGTGATAGGGCAAAGTAGATAATATGTAGGATTCACAATTTTTTTCTCCCCTAGCCTGAGAAA  chrX

561  GAGCATGGCAATTTTGATGAAATTTGGGGAAGTTCATTC  304-90_T7
     GAGCATGGCAATTTTGATGAAATTTGTACA-GTTCATTC  chrX
```

>307-90_SP6 vs. chr5: 134,432,514-134,433,030 (-), 517 bp overlap

```
  1  CCAGAATTTCAACTCTGGAAGGAGAAAACGAACTCCTGTAAGTTAATTTCTGATCTCCACAGGCAAGCGT  307-90_SP6
     CCAGAATTTCAACTCTGGAAGGAGAAAACGAACTCCTGTAAGTTAATTTCTGATCTCCACAGGCAAGCGT  chr5

 71  GTGCAGGAATGAACAGACACACATATGCATGCACACAAAATGAGGAAATTAAAAACACAATAAAGATGCA  307-90_SP6
     GTGCAGGAATGAACAGACACACATATGCATGCACACAAAATGAGGAAATTAAAAACACAATAAAGATGCA  chr5

141  ATTCCCATCTGCGCTCATTGGCATCATCTGCAGTTACGGGTGACAAGGAGCTTCACGGCACTGTCTCGCT  307-90_SP6
     ATTCCCATCTGCGCTCATTGGCATCATCTGCAGTTACGGGTGACAAGGAGCTTCACGGCACTGTCTCGCT  chr5

211  CCTCCTCCCTAACCCCCATCTCACCTGTGTCTGCATCATCATCAAACTGAGGTAGCCTCTTGCCCAGCTG  307-90_SP6
     CCTCCTCCCTAACCCCCATCTCACCTGTGTCTGCATCATCATCAAACTGAGGTAGCCTCTTGCCCAGCTG  chr5

281  GGGGAGGCCGGCATGGGGGTCATCCTGGAAGTTGTCATTCTCCAATGCCTCCAGCTGTCGGTTGATCCGC  307-90_SP6
     GGGGAGGCCGGCATGGGGGTCATCCTGGAAGTTGTCATTCTCCAATGCCTCCAGCTGTCGGTTGATCCGC  chr5

351  CGCTGCCTGGCTGCCCTGTCCAGCACCCGCCGCTGTCCAGGGTCCTGGGAGCGAACTAGATGGGGCAAAG  307-90_SP6
     CGCTGCCTGGCTGCCCTGTCCAGCACCCGCCGCTGTCCAGGGTCCTGGGAGCGAACTAGATGGGGCAAAG  chr5

421  AGAGTTACCGGGGTCAAGACAGAAGGAAAGTTGCACTGCTGTTGCCTAGAGCCCCAGTCTCCTCGCCTCG  307-90_SP6
     AGAGTTACCGGGGTCAAGACAGGAGGAAAGTTGCACTGCCGTTGCCTAGAGCCCCAGTCTCCTCGCCTCG  chr5

491  GCCCCTTCATTATGCTGTGAGGAATTC  307-90_SP6
     GCCCCTTCATTATGCTGTGAGGAATTC  chr5
```

>307-90_T7 vs. chr15: 78,792,690-78,792,809 (+), 120 bp overlap

```
  1  TTTCTGCCTCCTGTCCCCTCCTCTATCCCATCCTCCACATTGTCACCCCAAAGATCTCCTGACTCCTGCC  307-90_T7
     TTTCTGCCTCCTGTCCCCTCCTCTATCCCATCCTCCACATTGTCACCCCAAAGATCTCCTGACTCCTGCC  chr15

 71  CTCATCCACTGGGTCTGAATCCTTCCATGTCTCCCAACAACCACACCATG  307-90_T7
     CTCATCCACTGGGTCTGAATCCTTCCATGGCTCCCAACAACCACACCATG  chr15
```

>316-22B_T7 vs. chr7: 82,268,557-82,269,022 (-), 480 bp overlap

```
  1   CCACTAAACTTAGACACAAAGGAATTCTAGGGTAAAAATAAAAAAAGAAAATAATGAAAAGTATGAAAAA   316-22B_T7
      CCACTAAACTTAGACACAAAGGAATTCTAGGGTAAAAATAAAAATAGAAAATAATGAAAAGTATGAAAAA   chr7

 71   AATAAAAATAAGTTTTAAAAGGAAATGCCCCGAGAAAACATTATGAGATAGAAAAATTTCCAAAGATGTC   316-22B_T7
      A-TAAAAATAAGTTTTAAAAGGAAATGCCCCGAGAAAACATTATGAGATAGAAAAATCTCCAAAGATGTC   chr7

141   ATTGAATTAGGTTTCTCTAAGTTCATATGAGCTTTGATCATGTTGATTTAGAAAGCCTTATTTTCTTCAT   316-22B_T7
      ATTGAATTAGGTTTCTCTAAGTTCATATGAGCTTTGATCATGTTGATTTAGAAAGCCTTATTTTCTTCAT   chr7

211   GTCCTCCATTGTTTCTGAAATATTATATGTTCTACGATCTCTCTCTCTCTTTCCCTCCCTCTCTCTCTCT   316-22B_T7
      GTCCTCCATTGTTTCTGAA-TATTATATGTTCTACGATCTCTCTCTCTCTCTCTCTCTCTCTCTCTCTCT   chr7

281   CTCTCTCTCTCTGTGTGTGTGTGTGTGTGTGAGAGAGAGAGAGAGAGAAAGAGAGAGAGAGAGAGAGAGA   316-22B_T7
      CTCTCTCTCTGTGTGTGTGTGTGTGTGTGTGTGT----------------GTGTGTGAGAGAGAGAGAGA   chr7

351   GAGAGAGAGAGAGAGAGAGAGAGAGAGAGAGAGAGGTATGGCTATCAGTTTCTATATTTCTTTCTATCTT   316-22B_T7
      GAGAGAGAGAGAGAGAGAGAGAGAGAGAGAGGGAGGTATGGCTGTCAGTTTCTATATTTCTTTCTATCTT   chr7

421   CTGTAGAAG-----ATAGCTGATGTTGGCGTGAGCAAGGCACTGATCTATGAGCATAGCAGAATT   316-22B_T7
      CTGTAGAAAGAAACATCTCTGATGTTGGC-TGAGCAAGGCACTGATCTATGAGCATAGCAGAATT   chr7
```

>317-22A_SP6 vs. chr19: 50,281,939-50,282,430 (+), 509 bp overlap

```
  1   TGCCTTTTCTCCATGTTTCTCCTTACTCACACTTTACTCAACTCACCCCTGTGGAAGGAGCTTCTCTTAG   317-22A_SP6
      TGCCTTTTCTCCATGTTTCTCCTTACTCACACTTTACTCAACTCACCCCTGTGGAAGGAGCTTCTCTTAG   chr19

 71   CACCAGACAAGGACTATTATAGAAACCCAGAGAGCTGGTCAAAATGCAGAAAGCATATTGGGAAAAAGGA   317-22A_SP6
      CACCAGACAAGGACTATTATAGAAACCCAGAGAGCTGGTCAAAATGCAGAAAGCATATTGGGAAAAAGGA   chr19

141   TGGAGAGATAGAAAGTGCCAAAGGATCTGAAAATTGTGCCTCCTAGAAGTGACAGGGGCTTTTCACCCAA   317-22A_SP6
      TGGAGAGATAGAAAGTGCCAAAGGATCTGAAAATTGTGCCTCCTAGAAGTGACAGGGGCTTTTCACCCAA   chr19

211   GTTACCTCAAGAAACCTGAAGAGCGGGAACAAAACCTTAACAAGTACAATAGACATGCTAATGTGGTGGG   317-22A_SP6
      GTTACCTCAAGAAACCTGAAGAGCGGAAACAAAACCTTAACAAGTACAATAGACATGCTAATGTGGTGGG   chr19

281   GACACTTCACAGGATCCCTTCTTTAGACAAAGCTATATAGACCTCCAAGGAAAACCGAGACAGGGTTCCT   317-22A_SP6
      GACACTTCACAGGATCCCTTCTTTAGACAAAGCTATATAGACCTCCAAGGAAAACCGAGAGAGGGTTCCT   chr19

351   CCACAGGGAAAAGCATACTCATTAGTGAGACCGTAAAATATACAAAGCAGTAATATCTCTCTCTCTCTCT   317-22A_SP6
      CCACAGGGAAAAGCATACTCATTAGTGAGACCGTAAAATATACAAAGCAGTAATCTCTCTCTCTCTCTCT   chr19

421   CTCTCTCTCTCTCTCNCTCTCTCTCTCTCTCTCTCATGTGTGTGTGTGTGTGTGTGTGTGTGTGTGTGTG   317-22A_SP6
      CTCTCTCTCTCTCTCG---TGTGTGTGTGTGTGTG-TGTGTGTGTGTGTGTGTGTGTGAGAGAGAGAGAG   chr19

491   AGAGAGAGAGAGAGAGAGT   317-22A_SP6
      AGAGAGAGAGAGAGAGAGT   chr19
```

>317-22A_T7 vs. chr19: 50,386,642-50,387,194 (-), 553 bp overlap

```
  1   TTCCTCAGGAGAGAGTATGAGGAAGCCACTTCAAAGTCTTCACACAGTAGTGTCAAAGATAAGGAAAGCT   317-22A_T7
      TTCCTCAGGAGAGAGTATGAGGAAGCCACTTCAAAGTCTTCACACAGTAGTGTCAAAGACAAGGAAAGCT   chr19

 71   GGAAGAGGAGCAAGCAGACAGCTTGAGGGAGTCAAGGTAGTATTCTTTGAAGCCTCACCAGCGCAGGGAT   317-22A_T7
      GGAAGAGGAGCAAGCAGACAGCTTGAGGGAGTCAAGGTAGTATTCTTTGAAGCCTCACCAGCGCAGGGAT   chr19

141   GGGTGNCAAGTGTGGATGGGCTGCACCTTTAATGTTGAGGTAGAGCTGTGTCAAAATGCTTCAGTAAATA   317-22A_T7
      GGGTGGCAAGTGTGGATGGGCTGCACCTTTATTGTTGAGGTAGAGCTGTGTCAAAATGCTTCAGTAAATA   chr19

211   ACAGAGATTCCCACCCTGCTCCATCACTGACTCTCATCCGATCATTCTCTCGGTGGAAGAGGCTTCCTCT   317-22A_T7
      ACAGAGATTCCCACCCTGCTCCATCACTGACTCTCATCCGATCATTCTCTCGGTGGAAGATGCTTCCTCT   chr19

281   GTTCAGAGTGGGACACCTAGTTCTACCGAATAAAAATGAAGATAGATATCAGACAACCCGAGAAAAGATA   317-22A_T7
      GTTCAGAGTGGGACACCTAGTTCTACAGAATAAAAATGAAGATAGATATCAGACAACCAGAGAAAAGATA   chr19
```

```
351  AAACTGGGTCAACTGAAGGCATTAAATGCAGTGACATTTACTAAACTGACTGCGTTAACCTTTAGATCCA  317-22A_T7
     AAACTGGGTCAACTGAAAGCATTAAATGCAGTGACATTTACTAAACTGACTGTGTTAACCTTTAGATCCA  chr19

421  AAAGAAGGTAATGAAATAAAACAAACAAATTAGAAAGAAAATAAACATATCCACAGTCTTTANCAAATGG  317-22A_T7
     AAAGAAGGTAATGAAATAAAACAAACAAATTAGAAAGAAAATAAACATATCCACAGTCTTTATCAAATGG  chr19

491  ATCTATTTGGCAGGTGCCATAATTGAACGATTTTGCAGATATTTTGACAACTGGATAGAATTC  317-22A_T7
     ATCTATTTGGCAGGTGCCATAATTGAACGATCTTGCAGATATTTTGACAACTGGATAGAATTC  chr19
```

>319-20E_T7 vs. chr6: 143,502,805-143,503,360 (+), 556 bp overlap

```
  1  GAATTCACAAGGTCTAGGGATATGTTGTTGTAGGGACTGGAAGAAGCCTTAGTGAAAGTCTAAAGGAAGT  319-20E_T7
     GAATTAACAAGGTCTAGGGATATGTTGTTGTAGGGACTGGAAGAAGCCTTAGTGAAAGTCTAAAGGAAGT  chr6

 71  TTGGAATCTTGAGTGAGTGTGTATTGTTGACAGGGGCCAGTGCCTCAGAGCAGACGCTTCAGGGTATGCG  319-20E_T7
     TTGGAATCTTGAGTGAGTGTGTATTGTTGACAGGGGCCAGTGCCTCCGAACAGACACTCCAGGGTATGCG  chr6

141  CCTTTCCCATATGGCTTAGGCATGGAATTCACAAAGCCTTCCTTGGNACTGAGAGAAAATGTTGGTGGTC  319-20E_T7
     CCTTTCCCATATGGCTTAGGCATGGAATTCACAAAGCCTTCCTTGGGACTGAGAGAAAATGTTGGTGGTT  chr6

211  ATGTAAATTCAAGTGTTTCTGGATGACTACTTTGTGCAAAAACAAGGAATTGCAGCCTCCTCTTCCTGGA  319-20E_T7
     ATGTAAATTCAAGTGTTTCTGGATGACTACTGTGTGCAAAAACAAGGAATTGCAGCCTCCTCTTCCTGGA  chr6

281  TATTGACATTCTGAAACTGCTCACCTGCAGACATGGCTGTGACTATAAATTTCCCGTTGTGTGCTTTATA  319-20E_T7
     TATTTACATTCTGAAACTGTTCACCTGCAGACATGGCTGTGACTATAAATTCCCCATTGTGTGCTTTACA  chr6

351  AGTAAGTAGGCTGAGATTCTGGGTCATTATGGTAATTGGTGCAGCTCCATCAGAGTGCACACAGTCAACC  319-20E_T7
     AGTAAGTGGGCTGAGCTTCTGGGTCATTATGGTAATTGGTGCAGCTCCATCAGAGTGCACACAGTCAACC  chr6

421  TGAGCAGGTTTTTCTGAACATATGTCTATGTGTCTGTCTTTCCTTCATTCCTTGGCTGTCCCAGTCAGGT  319-20E_T7
     TGAGCAGGTTTTTCTGTACATATGTCTATGTGTCTGTCTTTCCTTCATTCCTTGGTTGTCCCAGTCAGGT  chr6

491  CTCTAGGACCAAGCCGTGCAGGTGGTGGTGCATAGTGAAGTGGGTGTATCTAACCCCTTGAACATT  319-20E_T7
     CTCTAGGACCAAGCCATGCAGGTGGTGGTGCATAGTGAAGTGGGTGTATCTAACCCCTTGAACATT  chr6
```

>320-4J_SP6 vs. chr13: 101,596,209-101,596,723 (+), 516 bp overlap

```
  1  GAATTCACTTTTGCCCTATATCTGTTCTGATGTTTCTTAGTTTGGGGTATAGTCCTAATATTTAGATTGC  320-4J_SP6
     GAATTCACTTTTGCCCTATATCTGTTCTGACGTTTCTTAGTTTGGGGTATAGTCCTAATATTTAGATTGC  chr13

 71  TGAGCTAACTTTTTAAAAGATTTTAAATTAAGACATAAGTCTTAGTTAGGGTTTCGTTGCTGTGAACAGA  320-4J_SP6
     TGAGCTAACTTTTTAAAAGATTTTAAATTAAGACATAAGTCTTAGTTAGGGTTTCGTTGCTGTGAACAGA  chr13

141  CTCCATGACCAAAGCAACTCTTACTAAGGACAGCATTTAATTGGCCCTGGCTTAACAGGTTCAGAGTTCA  320-4J_SP6
     CTCCATGACCAAAGCAACTCTTACTAAGGACAGCATTTAATTGGCCCTGGCTTAACAGGTTCAGAGTTCA  chr13

211  GTCCATTATCATCAAGGCAGGAACATGGCAGCATCCAGGCAGGCATGGTGCAGGAGGAGCTGCGAGTTCC  320-4J_SP6
     GTCCATTATCATCAAGGCAGGAACATGGCAGCATCCAGGCAGGCATGGTGCAGGAGTAGCTGCGAGTTCC  chr13

281  ACCTCTTGTTCCAAAGGCAGGTAGGAGAAGATTGGCTTCCAGGCAGCTAGGACAAGGGTCTTAAAGTCCA  320-4J_SP6
     ACCTCTTGTTCCAAAGGCAGGTAGGAGAAGATTGGCTTCCAGGCAGCTAGGACAAGGGTCTTAAAGTCCA  chr13

351  TACTCACTGTGACACACTTCCTCCAACAAGATCACACCTACTCCAACAAGACCACATCTCCAAATAGTGC  320-4J_SP6
     TACCCACCGTGACACACTTCCTCCAACAAGATCACACCTACTCCAACAAGACCACATCTCCAAATAGTGC  chr13

421  TATTCCCTGAGCCAAGCATATTCAAACCACACAACATAATTGCAATAATTTCTCTCTTCCCTTTTTCCCT  320-4J_SP6
     TATTCCCTGAGCCAAGCATATTCAAACCACACAACATAATTGCA-TAATTTCTCCCTTCCCTTTTTCCCT  chr13

491  CCAAGGCTCCCATATACCCTCCACTC  320-4J_SP6
     CCAAGGCTCCCATATACCCTCCACTC  chr13
```

>320-4J_T7 vs. chr13: 101,703,433-101,704,061 (-), 636 bp overlap

```
  1 CTCCATGGAAAGAGAGGCTACATGGTCTCCACAAGACTTATAAAGTTTATACAGCAGTGACCTCCATCCC  320-4J_T7
    CTCCATGGAAAGAGAGGCTACATGGTCTCCACAAGACTTATAAAGTTTATACAGCAGTGACCTCCATCCC  chr13

 71 CTGAGTCCTTTCCCAAAGAGTCATGTAGCCATTTATTCTGGAAACCATAAAATAGGAAAAGGGGAATTTC  320-4J_T7
    CTGAGTCCTTTCCCAAAGAGTCATGTAGCCATTTATTCTGGAAACCATAAAATAGGAAAAGGGGAATTTC  chr13

141 TACATCTTCCAGCAGCGCCGGGGACAGAATCTGAGCAGACACTGCTGTTCTGATATTGTTCTTCTGTGCA  320-4J_T7
    TACATCTTCCAGCGGCCCCGGGGACAGAATCTGAGCAGACACTGCTGTTCTGATATTGTTCTTCTGTGCA  chr13

211 ACAGGGTTTGGAAGCAGACTTAACTCATGGTGTGCCAACTGCTTTTATGGGACTTCTTTCTCCAATTCTC  320-4J_T7
    ACAGGGTTTGGAAGCAGACTTAACTCATGGTGTGCCAACTGCTTTTATGGGACTTCTTTCTCCAATTCTC  chr13

281 AAACCTATCAGTGGAATGGATGTACTTAGCAGTCGGCAGCAGCAGCACTT-GGTTCTTAGCCTGGAGAGG  320-4J_T7
    AAACCTATCAGTGGAATGGATGTACTTAGCAGTCGGCAGCAGCAGCACTTTGGCTCTTAGCCTGGAGAGG  chr13

350 AAGAGGTAATGTATTTGGGGGGAAAAAAAAGACCGAGTAAATATTCCAGTAACCATTAACTTTCTATTCT  320-4J_T7
    ------TAATGTATTTGGGGGGAAAAAAA-GACCGAGTAAATATTCCAGTAACCATTAACCTTCTATTCT  chr13

420 CAGCGAGATCAAATCATTGCTACGTAAGGGTGATGGTGGAGATACAGGGTTCCTTTGGTGGATCGTAGCA  320-4J_T7
    CAGCGAGATCAAATCATTGCTAAGTAAGGGTGATGGTGGAGATACAGGGTTCCTTTGGTGGATCCTAGCA  chr13

490 GGGGCAGCAGATACATTGCTGCCAGCAGCCCCAGTGGGAACTGCTGTGCCAGTGTGTTCTCTCTACAACA  320-4J_T7
    GGGGCAGCAGATACATTGTTGCCAGCAACCCCAGTGGGAACTGCTGTGCCAGTGTGTTCTCTCTACAACA  chr13

560 GCTTAAAACAGTCTCAGTTATGTATATTGTGAGGAGCTAACCCAGTTACCCAATCTTTCCCTCTTGTGGG  320-4J_T7
    GCTTAAAACAGTCTTAGTTATGTATATTGTGAGGATCTAACCCAGTTACCCAATCTTTCCCTCTTGTGGG  chr13

630 GATAATA  320-4J_T7
    GA-AATA  chr13
```

>322-4J_SP6 vs. chrX: 130,761,839-130,762,372 (+), 525 bp overlap

```
  1 GAATTCATTCTGTATACCAGGCTGGCCTAGAACTCAGAAATCCACCTGCCTCTGCCTCCCGAGTGCTGGG  322-4J_SP6
    GAATTCATTCTGTAGACCAGGCTGGCCTAGAACTCAGAAATCCACCTGCCTCTGCCTCCCGAGTGCTGGG  chrX

 71 ATTAAAGGCGTGCGCCACCACGCCCGGCTCAATTTATCTTTTCAGTAATTCATTTGTCTCCATCACACAC  322-4J_SP6
    ATTAAAGGCGTGCGCCACCACGCCCGGCTCAATTTATCTTTTCAGTAATACATTTGTCTCCATCACACAC  chrX

141 ATTCATCCTGTCTCTTACATTTCCATATTTTGAAATTTTTTGTTGACTATGAAGTGTATTTTTTTT-ACA  322-4J_SP6
    ATTCATCCTGTCTCTTACATTTCCATATTTTGACATTTTTTGTTGACTATGAAGTGTATTTTTTTTTACA  chrX

210 TAATTCATGTGCTGGTCCCATCGCACCAAGTCTTATTGATACCCACAGAATCATATTCACCATTGCAATC  322-4J_SP6
    TTATTCATGTGCTGGTCCCATCGCACCAAGTCTTATTGATTCCCACAGAATCATATTCACCATTGCAATC  chrX

280 TTAGAAACTGAGTTCAGTCCCCAGGCTTGCTGGCCAGACAACCTAGCCCACTTGATTAGTTCCACACCAC  322-4J_SP6
    TTAGAAACTGAGTTCAGTCCCCAGGCTTGCTGGCCAGACAACCTAGCCCACTTGATTAGTTCCACACCAC  chrX

350 TGAGAGACTCTATATCAGTAAAACAATAATAGACAGAGCCTGAAGAATGACACCCAGGAGAGTC-CTCTG  322-4J_SP6
    TGAGAGACTCTATATCAGTAAAAAAATAATAGACAGAGCCTGAAGAATGACACCCAGGAGTGACACACAC  chrX

419 GCCTACA-------ACACACACACACACATATACACACAAATAAATAAGAATGATTTTGAAATAAAAATT  322-4J_SP6
    ACACACACAGACACACACACACACACACATATACACACAAATAAATAAGAATGATTTTGAAATAAAAATT  chrX

482 CCAATACTAGCACAAAGAACACTCTTCCCTTTTTCATATATATT  322-4J_SP6
    CCAATACTAGCACAAAGAACACTCTTCCCTTTTTCATATATATT  chrX
```

>322-4J_T7 vs. chrX: 130,878,933-130,879,529 (-), 602 bp overlap

```
  1 ACAC-CACACACACACACACACACACACACACATGCATGCAAGCACACACACACATTCATGTGAGCAC  322-4J_T7
    ACACACACACACACACACACACACACACACACACATGCATGCAAGCACACACACACATTCATGTGAGCAC  chrX

 70 ACATACACACACACAGGGGAGAGAGAGAGAGAGAGGGAGAGAGAGAGAGAGAGAGAGAGAGAGAGAGAGA  322-4J_T7
    ACATACACACACAG--------------------GGGAGAGAGAGAGAGAGAGAGAGAGAGAGAGAGAGA  chrX
```

```
140  GAGAGAGAGAGAGAGAGAGAGAGAGAGAGAAATCATAAATATGTTCCAGTATCATTCAGCCTAAAACAACAC  322-4J_T7
     GAGAGAGAGAGAGAGAGAGAGAGAGAGAGAAATCATAAATATGTTCCAGTATCATTCAGCCTAAAACAACAC  chrX

210  CATTTCCTTTCAAACAAATATTTCTCTGACCTTTATTAAACCCACTTTGACTGAATTAATTCTGCTTCTA  322-4J_T7
     CATTTCCTTTCAAACAAATATTTCTCTGACCTTTATTAAACCCACTTTGACTGAATTAATTCTGCTTCTA  chrX

280  CTGCACAACAGCTTGTAAGGGGGTTGGGGTGGGGAGAAAGGAGGGG-AATGAAGCAGAAGTGAGAGGAGG  322-4J_T7
     CTGCACAACAGCTTGTAAGGGGGTTGGGGTGGGGAGAAAGGAGGGGGAATGAAGCAGAAGTGAGAGGAGG  chrX

349  CAGAAAGAAAGGGAGAAGAAAGGGAGGAGAAAGAAGAACAAGGTGCTGACAATGTAAGAAATGATGTTTT  322-4J_T7
     CAGAAAGAAAGGGAGAAGAAAGGGAGGAGAAAGAAGAACAAGGTGCTGACAATGTAAGAAATGATGTTTT  chrX

419  GCTACAACCATAGGAATATTAAAAACACATTAGAATGATGTTTATAGATCACATATGTTAACATTGAAAA  322-4J_T7
     GCTACAACCATAGGAATATTAAAAACACATTAGAATGATGTTTATAGATCACATATGTTAACATTGAAAA  chrX

489  GCATTCATAGTATCTTTTAATATAATTTGTTTGTTTTAATTTTATGTGCATTGGTGTTTTGCCTGCATGT  322-4J_T7
     GCATTCATAGTATCTTTTAATATAATTTGTTTGTTTTAATTTTATGTGCATTGGTGTTTTGCCTGCATGT  chrX

559  GCATGTCCGTGTGAGGGTGTCTGATTCCCTGAGTCTGT-GAATTC  322-4J_T7
     GCATGTCCGTGTGAGGGTGTCTGATTCCCTGGAACTGTTGAATTC  chrX
```

>327-5I_SP6 vs. chr7: 130,719,661-130,720,069 (-), 409 bp overlap

```
  1  AAGGAGTCCCAGGATGTGGGCTTCCCTGGGTCTCTCTCAGGGCATTGCCTCCTATGATGCTCTGGTGCAG  327-5I_SP6
     AAGGAGTCCCAGGATGTGGGCTTCCCTGGGTCTCTCTCAGGGCATTGCCTCCTATGATGCTCTGGTGCAG  chr7

 71  GTGGAGGATGCAGTGTTGGACACCTATGACTTCGTGTATGATCAGGCAATGAAGAGCCCATCTAGCAACT  327-5I_SP6
     GTGGAGGATGCGGTGTTGGACACCTATGACTTCGTGTATGATCAGGCAATGAAGAGCCCATCTAGCAACT  chr7

141  GGTGGCAGGAGCTGGCCATCATCCAGGACACGGTGAGCTAAGGACAGGGTAGTCATACTTGCTGTGCTCC  327-5I_SP6
     GGTGGCAGGAGCTGGCCGTCATCCAGGACACGGTGAGCTAAGGACAGGGTAGTCATACTTGCTGTGCTCC  chr7

211  AGAGAAAGGATGCCAGAAATCCTGTAGAGTCAGCCATCCTGGCAAGGCTTCAAGAGGAGGTCTCAGAACG  327-5I_SP6
     AGAGAAAGGATGCCAGAAATCCTGTAGAGTCAGCCATCCTGGCAAGGCTTCAAGAGGAGGTCTCAGAACG  chr7

281  CAGGTTCATTTTGAGTAGATGACTGAGCTCCCACCCATCCCCCTCCACCCCAAATAGGACTGGCTACCCA  327-5I_SP6
     CAGGTTCATTTTGAGTAGATGACTGAGCTCCCACCCATTCCCCTCCACCCCAAATAGGACTGGCTACCCA  chr7

351  ATGTATGGCTAATGCAAAATAAAAGGGCAATGCCCCTGTTCAGTAGGGACTGAGAATTC  327-5I_SP6
     ATGTATGGCTAGTGCAAAATAAAAGGGCAATGCCCCTGTTCAGTAGGGACTGAGAATTC  chr7
```

>327-5I_T7 vs. chr7: 130,587,753-130,588,368 (+), 616 bp overlap

```
  1  GAATTCTTTTTATTATGAGGAAGGCCTTAGGCTTCAGTGAGAATCAGTGTGTGGCTATAGGCCACAGTGA  327-5I_T7
     GAATTCTTTTTATTATGAGGAAGGCCTTAGGCTTCAGTGAGAATCAGTGTGTGGCTATAGGCCACAGTGA  chr7

 71  AAACTTTGGACTGCCCCACAAAGCTGATCAGCCTTGAAGAGGTAGGCCTAGGACTGTGGTAAGGAACTTA  327-5I_T7
     AAACTTTGGACTGCCCCACAAAGCTGATCAGCCTTGAAGAGGTAGGCCTAGGACTGTGGTAAGGAACTTA  chr7

141  GAGTAAACTTAAGAGTGTGAAGAACCTCAGCATAACAACTTTTGATACAAGAACCTACCTTCTTGCTCAT  327-5I_T7
     GAGTAAACTTAAGAGTGTGAAGAACCTCAGCATAACAACTTTTGATACAAGAACCTACCTTCTTGCTCAT  chr7

211  GACACTTGCTAAGGACACTCATAATGTCATTCCTAAGATTCTAACTTGAATCTAAGGTTTTCTTGATATT  327-5I_T7
     GACACTTGCTAAGGACACTCATAATGTCATTCCTAAGATTCTAACTTGAATCTAAGGTTTTCTTGATATT  chr7

281  CTCATTGGTGTATAAACTTTTCCCCAGGATTTCTTTGCGGTATAATGTCTTCTTGAGTACTGACTGTGCC  327-5I_T7
     CTCATTGGTGTATAAACTCTTCCTTAGGATTTCTTTGCGGTATAATGTCTTCTTGAGTACTGACTGTGCC  chr7

351  TGAGGTCTTTCCCAGATTCACATTGGGGTCTAAGGCTTTCCCCTGGTTCTGTCTCATAAGGCCTTTTTAT  327-5I_T7
     TGAGGTCTTTCCCAGATTCACATTGGGGTCTAAGGCTTTCCCCTGGTTCTGTCTCATAAGGCCTTTTTAT  chr7

421  CCTCTCACTTGTCATTCAAATATATTTAGGGTTTCTCTGGAACACAGGGTCCTCCTCAGGTACTCAGAAC  327-5I_T7
     CCTCTCACTTGTCATTAAAATATATTTAGGGTTTCTCTGGAACACAGGGCCCTCCTCAGGTACTCAGAAC  chr7
```

```
491  TCTACAGAGGCCTTCCTCAGGTTGTAATTAGGGCCTAAGGCCTTCTCAGCATCTCACTGGGACCTAAGAC  327-5I_T7
     TCTACAGAGGCCTTCCTCAGGTTGTAATTAGGGCCTAAGGCCTTCTCAGTGTCTCACTGGGACCTAAGAC  chr7

561  CTTCCCCTGCATCTCACTTGTGTCTAAGGACATTCTAAAGTTCTAATTGGCACCTA  327-5I_T7
     CTTCCCCTGCATCTCACTTGTGTCTAAGGACATTCTAAAGTTCTAATTGGCACCTA  chr7
```
